# Risk factors of neuroblastoma: a systematic review and meta-analysis

**DOI:** 10.3389/fpubh.2025.1576101

**Published:** 2025-06-25

**Authors:** Felix M. Onyije, Roya Dolatkhah, Ann Olsson, Liacine Bouaoun, Joachim Schüz

**Affiliations:** Environment and Lifestyle Epidemiology Branch, International Agency for Research on Cancer (IARC/WHO), Lyon, France

**Keywords:** neuroblastoma, high birthweight, Cesarean section, breastfeeding, pesticides, systematic review and meta-analysis

## Abstract

**Introduction:**

Neuroblastoma (NB) is the most common extracranial tumor in children. Synthesizing and elucidating modifiable risk factors is fundamental to inform primary prevention of NB. The objective is to review literature and synthesize risk factors for NB.

**Methods:**

PubMed, Web of Science, and Embase databases were searched using lists of key words and MeSH terms related to exposures and risk of NB. Studies were included if they were case-control or cohort studies of children under the age of 20 years at diagnosis and reported Relative Risks (RRs) with 95% confidence intervals (CIs). Pooled effect sizes (ES) and 95% CIs for risk factors associated with NB were estimated using random-effects models.

**Results:**

We included 50 eligible studies from Asia, Europe, and North America, and Oceania on cases of NB diagnosed between 1964 and 2016. We observed associations for maternal occupational exposure to pesticides during preconception/pregnancy (ES 1.62, CI 1.04–2.54), high birthweight [(>4,000 g) ES 1.21, CI 1.02–1.42], and Cesarean section (ES 1.14, CI 1.00–1.30) and the risk of NB. Parental smoking showed a weak association, while breastfeeding ≥6 months (ES 0.50, CI 0.30–0.84) was inversely associated with NB. Birth characteristics such as low birthweight (<2,500 g), small and large-for-gestational age, gestation age <37 weeks and gestation age >40 weeks, and assisted reproductive technology were not associated with NB. Similarly, no associations were suggested for parental age, gestational diabetes, and pre-eclampsia. Maternal alcohol consumption during preconception/pregnancy, maternal intake of vitamin and folic acid during pregnancy, paternal occupational exposure to extremely low-frequency magnetic fields (ELF-MF), and maternal X-ray exposure during pregnancy were also not associated with the risk of NB. Paternal occupational and child's postnatal exposure to pesticides were also not associated with NB.

**Discussion:**

This systematic review and meta-analysis suggest that maternal occupational exposure to pesticides during preconception/pregnancy, high birthweight, Cesarean section, and breastfeeding (beneficial) were associated with the risk of NB, but all associations were rather modest in strength. Synthesizing of these risk factors are needed to inform whether there are avenues for primary prevention of NB.

## 1 Introduction

Neuroblastoma (NB) is the most common extracranial tumor in children and the most frequent solid malignancy in children under 1 year (1). Approximately 60% of NB occur before age 2 and about 97% are diagnosed before the age of 10 years (2, 3). Globally, the incidence pattern of NB is unique among the childhood cancers and varies greatly across age groups. In developed countries, NB accounts for annually 11–13 per million in children aged <15 years and 65 per million in children <1 year but only 1 per million in children of 10–14 years (4).

Like other common childhood cancers, NB is heterogeneous, and it is classified into different risk strata such as low-risk, intermediate-risk, and high-risk groups. Survival rate varies by risk groups, and is higher than 95% in the low-risk group whereas only around 50% in the high-risk group (5). However, it is known that some of the NB patients are undergoing spontaneous regression even without any form of treatment, a more common phenomenon with NB but observed to a lesser extent in other few cancer types like renal cell carcinoma, malignant melanoma, choriocarcinoma and lymphoid malignancies (6).

While some individual epidemiological studies have suggested some risk factors associated with NB, overall its etiology remains largely unknown. These include paternal smoking, maternal alcohol consumption during the preconceptional period or pregnancy, childhood exposure to pesticides, Cesarean section (C-section), and high birthweight exceeding 4,000 g (3, 7–10). However, the evidence is inconsistent as there are also studies that have shown no associations for the same risk factors (11–15). Thus, to date no modifiable risk factor for NB has been clearly established.

NB has a variety of clinical behaviors that are mostly influenced by the biology, including unique abilities to suppress the host immune system. Chromosomal aberration is frequent in NB. For example, deletions of the short arm of chromosome 1 (1p) occur in about 70% of advanced stage. However, it is still unclear whether these events are responsible for the initiation of NB (16–18). While biology undoubtedly plays a central role, modifiable exposures could influence the timing of disease onset, immune system priming, or epigenetic regulation (19). In our study, we have been careful to avoid strong causal claims and instead frame our findings as associations that warrant further mechanistic exploration. Therefore, the aim of this systematic review and meta-analysis was to synthesize and elucidate evidence from different epidemiological studies. To give a consolidated overview of risk factors potentially associated with NB which may inform primary prevention of the disease.

## 2 Methods

### 2.1 Search strategy and study selection

This systematic review and meta-analysis was conducted according to the 2020 Preferred Reporting Items for Systematic Reviews and Meta-Analyses (PRISMA) checklist (20) (Supplementary Table 1, p. 3). The search strategy used for article selection and methods for data extraction and analysis have been previously published (21, 22). We searched PubMed, Web of Science, and Embase databases with no restriction on publication date but selected articles are all written in English language. Identified peer reviewed articles were retrieved, imported, and screened for duplicates in EndNote version X9.3.3. The authors, FMO and RD assessed the titles, abstracts, and full text of the articles independently to determine their eligibility (Supplementary Table 2, p. 6) (23), differences arising from the independent selection process were resolved by seeking opinion of the third author, AO. Additional articles were sourced from lists of references. The search strategy was structured in line with Population, Exposure, Comparator and Outcome (PECO) components and included a list of key words and MeSH terms (Supplementary Tables 3–5, p. 7–13). The search was initially conducted in June 2022 and subsequently updated until January 2025. The studies were included if they were case-control or cohort studies of childhood NB under the age of 20 years, we reported exposure time windows, and provided estimates of Relative Risks (RRs) such as Odds Ratio (OR), Hazard Ratio (HR), Standardized Mortality Ratio (SMR), Mortality Rate Ratio (MRR), Standard Incidence Ratio (SIR), or Incidence Rate Ratio (IRR) with 95% confidence intervals (CIs). We checked publications from the same region for overlaps of their study populations. The inclusion and exclusion criteria were defined a priori (Supplementary Table 2, p. 6) (21, 22).

### 2.2 Data extraction

Risk factors extracted included birth and parental characteristics, environmental and occupational exposures pesticides, radiation, and lifestyle exposures. Exposure time period such as preconceptional, prenatal and postnatal were also considered. Other information extracted includes authors' name, year of publication, study location, period and age range of diagnosis, exposure assessment methods, outcome ascertainment, number of NB cases and controls or, if not available, the study population, follow-up duration, and risk estimates with their respective 95% CIs. Information regarding study design (case-control and cohort or registry-based case-control) was also extracted. Registry-based case-control studies were considered as cohort studies in the present analysis (21). Case-control studies are thereby studies requiring interaction with the study participants.

### 2.3 Quality assessment of eligible articles

All eligible articles underwent a quality assessment of their methodological quality using the Joanna Briggs Institute (JBI) critical appraisal tools for case-control and cohort studies (24). The appraisal checklist has 10 criteria for case-control and 11 for cohort studies. Every question answered with a “yes” received a score of 1, while a “no” scored 0, and “unclear” or “not applicable” received also 0 (Supplementary Tables 6, 7, p. 14–15). Prior to the critical appraisal of the articles, we systematically checked the articles for overlaps of their study populations and by risk factors.

### 2.4 Statistical analyses

We performed random-effects meta-analyses in order to estimate pooled effect sizes (ES) with their respective 95% CIs. Funnel plots and Egger's test were employed to assess potential publication bias (25). The *I*^2^ statistic was calculated to quantify the heterogeneity of the results between studies. *I*^2^ values of 0% were considered to represent “no heterogeneity”, from 1 to 35% “low heterogeneity”, from 36 to 55% as “moderate”, from 56 to 70% as “substantial” and above 71% as “considerable” heterogeneity (26). Analyses were conducted both combining case-control and cohort studies, and separately by study design (case-control vs. cohort studies). The combined analysis is presented as the primary focus, unless otherwise stated. Analyses were conducted using STATA^®^ software, version 15.1 (College Station, TX, USA) using a nominal significance level of 0.05.

## 3 Results

### 3.1 Study characteristics

**Figure 1:**
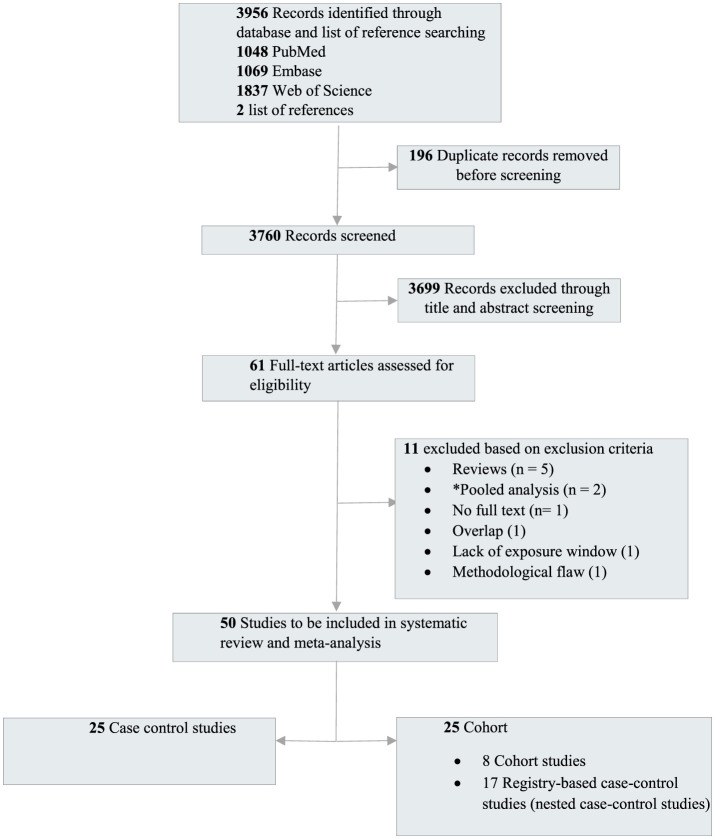
Preferred reporting items for systematic reviews and meta-analyses flow diagram outlining the study selection. *The pooled studies were excluded as the primary studies were already published and included in our meta-analysis.

A total of 3,760 unique records were retrieved and screened, leading to the evaluation of 61 full texts. Among these, 50 studies [25 case-control and 25 cohort studies (including registry-based nested case-control studies)] met the study inclusion criteria (Figure 1 and Table 1).

**Table 1:**
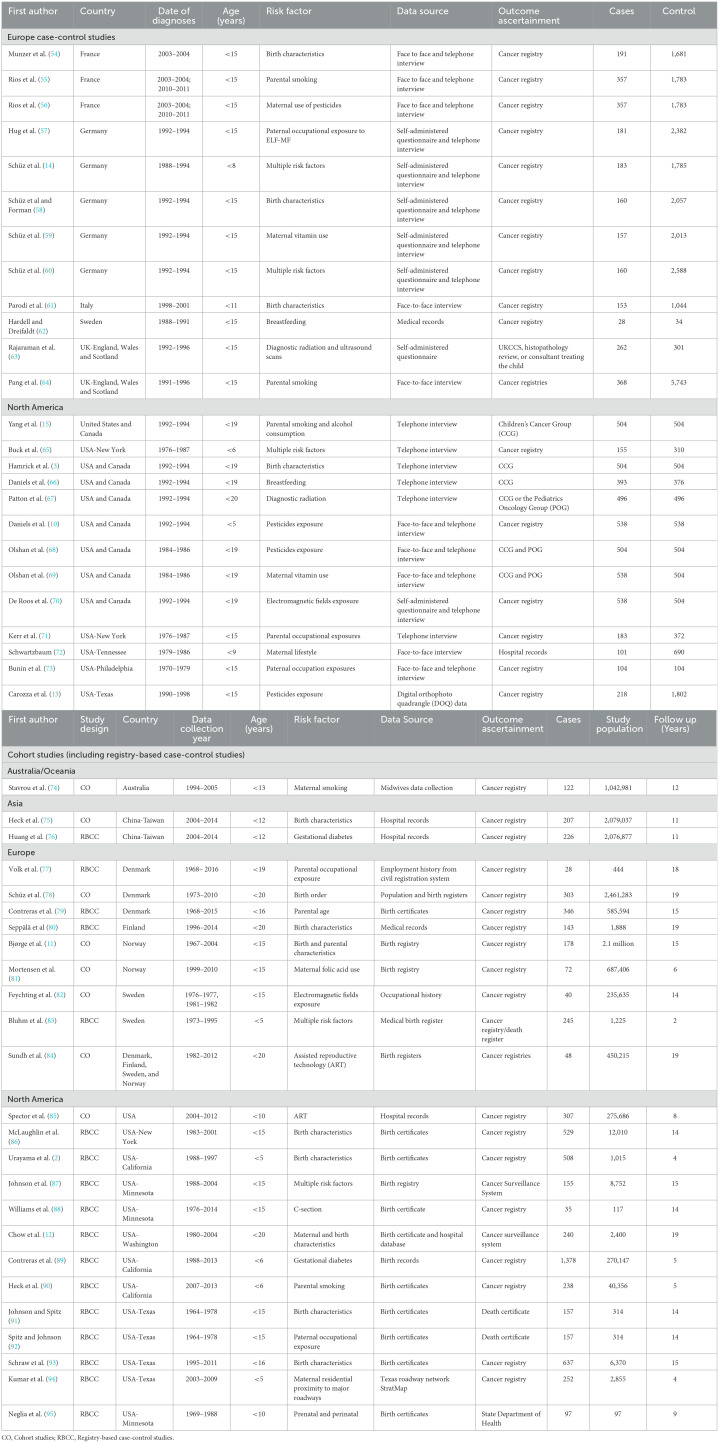
Characteristics of the 50 studies included in the systematic review and meta-analysis, sorted by country.

CO, Cohort studies; RBCC, Registry-based case-control studies.

Among all the studies that met the criteria, 52% (*n* = 26) were carried out in North America. Europe came next with 42% (*n* = 21), followed by Asia with 4% (*n* = 2), and Oceania with 2% (*n* = 1). There were no eligible studies in Latin America and Africa.

### 3.2 Study bias and quality assessment

The 50 articles critically appraised for quality using the JBI tools were generally of good quality (86%). The least ranked case-control study scored 7 out of 10 points, while for cohort study, it was 7 out of 11 points. Thus, all screened articles appraised were included in the final analysis (Supplementary Tables 6, 7, p. 14–15).

### 3.3 Birth and parental characteristics

**Figure 2:**
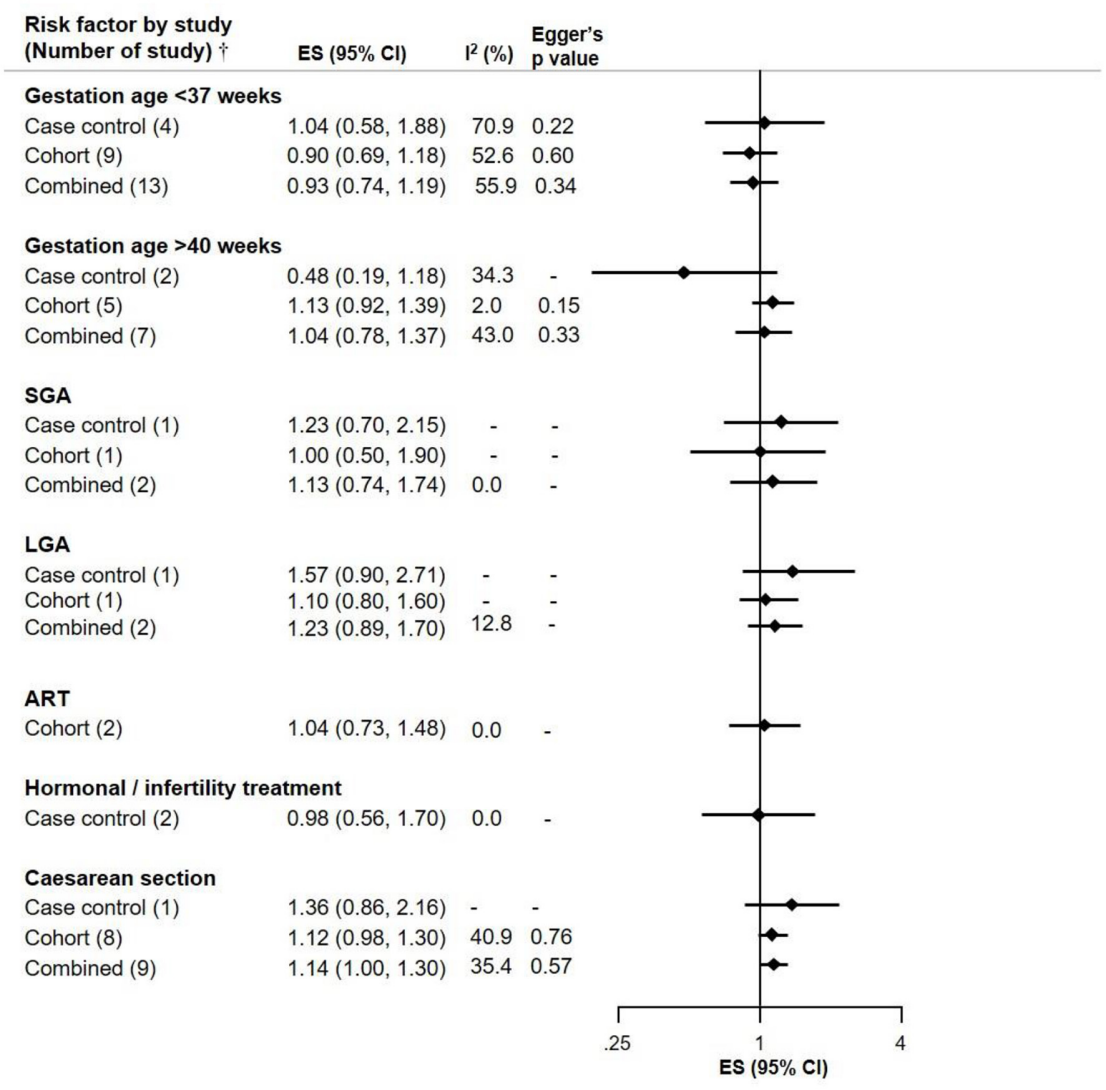
Meta-analysis of pooled effect sizes (ES) of exposure to birth characteristics [Gestation Age <37 weeks, >40 weeks; Small for Gestation Age (SGA), Large for Gestation Age (LGA); assisted reproductive technology (ART); Hormonal/Infertility treatment and C-section] for the risk of NB and heterogeneity (*I*^2^) with Eggers *p*-value by study design.^†^Where only one study was identified, it is referred to as RR and not ES.

C-section was a suggestive risk factor of NB (ES 1.14, CI 1.00–1.30) with Eggers *p*-value of 0.57 and moderate heterogeneity across studies. This outcome is based on 8 cohort studies and one case-control study. There was no association observed between gestational age <37 weeks or > 40 weeks and NB risk. Analyses relating to small and large for gestational age were also not suggestive of association with NB, though the ES for large gestational age (LGA) was slightly elevated with confidence intervals including 1 (ES 1.23, CI 0.89–1.70) based on 1 cohort and 1 case control study. Assisted reproductive technology (ART) and hormonal/infertility treatment did not show an association with NB (Figure 2).

**Figure 3:**
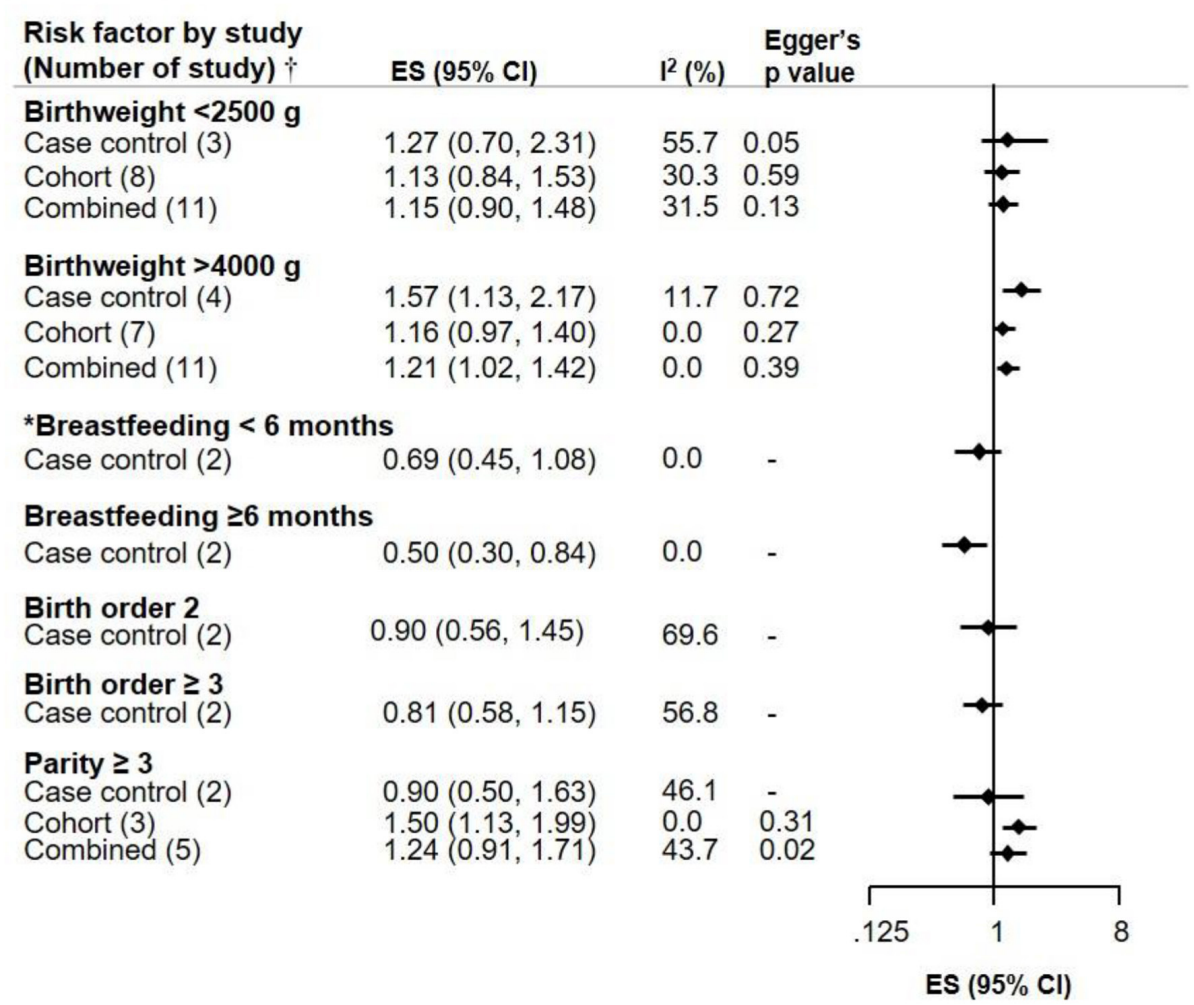
Meta-analysis of pooled effect sizes (ES) of exposure to birth characteristics (Birthweight, Breastfeeding, Birth order 2 and ≥3, and Parity ≥3) for the risk of NB and heterogeneity (*I*^2^) with Eggers *p*-value, by study design.^†^Where only one study was identified, it is referred to as RR and not ES. *Breastfeeding <6 months does not include 0 months.

Low birthweight (<2,500 g) was not associated with NB, but an association was seen between high birthweight (>4,000 g) and the risk of NB (ES 1.21, CI 1.02–1.42). Breastfeeding appeared to be inversely associated with NB in an exposure-response manner, as shown in children breastfed for ≥6 months (ES 0.50, CI 0.30–0.84). Birth order (2 and ≥3) was not associated with NB risk. While the mother's parity ≥3 showed an increased risk of NB based on three cohort studies (ES 1.50, CI 1.13–1.99), this was attenuated when combined with 2 case control studies, and there was moderate heterogeneity across studies (Figure 3).

**Figure 4:**
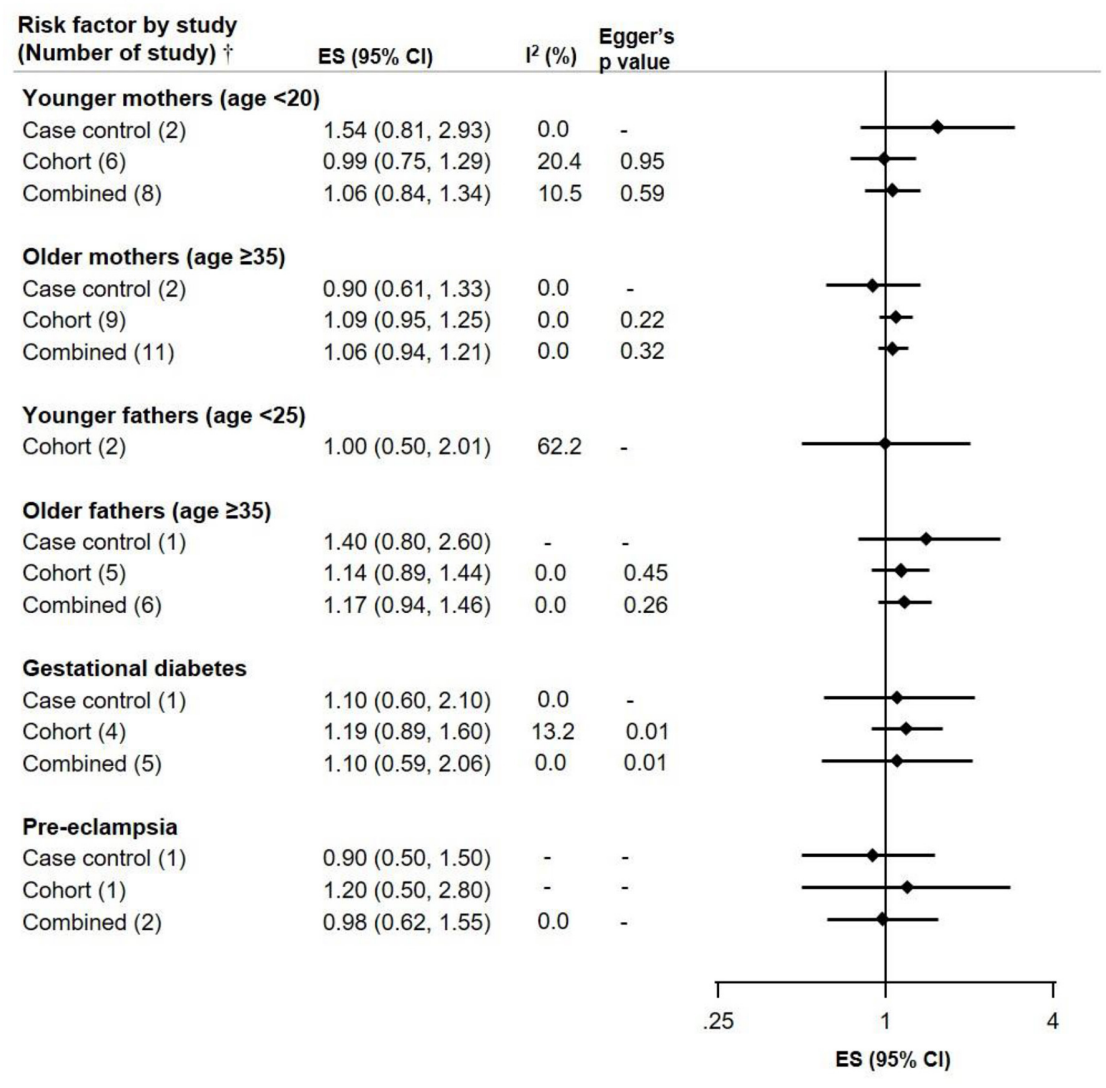
Meta-analysis of pooled effect sizes (ES) of exposure to parental characteristics [Younger mothers (age <20) and fathers (age <25)], Older mothers and fathers (age ≥35), Gestational diabetes and Pre-eclampsia for the risk of NB and heterogeneity (*I*^2^) with Eggers *p*-value, by study design.^†^Where only one study was identified, it is referred to as RR and not ES.

For young mothers (<20 years) and older fathers (≥35 years) the ES were slightly elevated in two case-control studies (ES 1.54, CI 0.81–2.93 and ES 1.40, CI 0.80–2.60), but not in the more numerous cohort studies; the potential selection bias leading to spurious associations with young parental age in childhood cancer has been noted before (27). Gestational diabetes, and pre-eclampsia were not associated with NB risk. However, Egger's *p*-value was 0.01 for the cohort and combined studies on gestational diabetes, suggesting potential publication bias (Figure 4).

### 3.4 Lifestyle

Maternal smoking during pregnancy (ever smokers) showed a weak association for the risk of NB. Similarly, there was a weak association for mothers who smoked 1–10 cigarettes per/day in case-control studies (ES 1.35, CI 1.00–1.83), but it was attenuated when combined with the one cohort study on the topic (ES 0.85, CI 0.50–1.47; Table 2). In the same vein, paternal smoking during preconception/prenatal showed weak association with NB (ES 1.12, CI 0.97–1.30, *p* = 0.44), based on 4 case-control studies with no heterogeneity across studies. Maternal consumption of alcohol during preconception/pregnancy did not show an association with NB risk. Likewise, no association was observed for maternal intake of vitamin and folic acid during preconception/pregnancy and the risk of NB (ES 0.98, CI 0.53–1.84, 2 case-control and 1 cohort studies), although with substantial heterogeneity.

**Table 2:** Meta-analysis of pooled effect sizes (ES) of exposure to lifestyle, pesticides, and radiation for the risk of NB and heterogeneity (*I*^2^) between studies, by study design.

**Risk factor**	**Window**	**Case-control**						**Cohort**						**Combined**					
	**Period**	***N***	**ES** ^†^	**LCI**	**UCI**	*I*^2^ **(%)**	**Egger's** ***p*** **value**	***N***	**ES** ^†^	**LCI**	**UCI**	*I*^2^ **(%)**	**Egger's** ***p*** **value**	***N***	**ES** ^†^	**LCI**	**UCI**	*I*^2^ **(%)**	**Egger's** ***p*** **value**
**Lifestyle**																			
Maternal smoking	Prenatal	4	1.19	0.98	1.45	0.0	0.44	2	1.08	0.71	1.64	0.0	-	6	1.17	0.98	1.40	0.0	0.42
Maternal smoking 1–10/day	Prenatal	3	1.35	1.00	1.83	0.0	0.65	1	0.85	0.50	1.47	-	-	4	1.21	0.93	1.57	0.0	0.56
Paternal smoking	Preconception/prenatal	4	1.12	0.97	1.30	0.0	0.44												
Maternal alcohol	Preconception/prenatal	4	0.90	0.76	1.07	0.0	0.87												
Maternal vitamin and folic acid intake	Prenatal	2	0.95	0.39	2.07	91.1	-	1	1.05	0.53	2.06	-	-	3	0.98	0.53	1.84	82	0.36
**Pesticides**																			
Child's exposure to general pesticides	Postnatal	2	1.28	0.73	2.27	66.8	-												
Maternal occupational exposure to general pesticides	Preconception/prenatal	4	1.62	1.04	2.54	17.5	0.84												
Paternal occupational exposure to general pesticides	Preconception/prenatal	5	1.09	0.65	1.83	24	0.35												
**Radiation**																			
Maternal exposure to X-rays	Prenatal	3	1.11	0.77	1.59	0.0	0.65												
Paternal exposure to ELF-MF >0.15–0.2 μT	Preconception/prenatal	2	0.86	0.64	1.16	0.0	-	1	1.80	0.60	5.30	-	-	3	0.91	0.68	1.21	0.0	0.08
Paternal exposure to ELF-MF >0.2 μT	Preconception/prenatal	2	0.99	0.74	1.32	0.0	-	1	0.90	0.20	3.60	-	-	3	0.99	0.74	1.30	0.0	0.79
Maternal residential proximity to major roads (< 500 m)	Preconception/prenatal	1	1.23	0.91	1.67	-	-												

^†^Where only one study was identified, it is referred to as RR and not ES.

### 3.5 Chemicals and radiations

Exposure to general pesticides during childhood and the risk of NB, showed a slightly elevated ES but with wide confidence intervals (ES 1.28, CI 0.73–2.27) based on 2 case-control studies (Table 2). On the other hand, we observed an association for maternal exposure to general pesticides during preconception/pregnancy and the risk of NB (ES 1.62, CI 1.04–2.54; four case-control studies). We did not observe an association for paternal exposure to general pesticides during preconception/pregnancy and the risk of NB (ES 0.86. CI 0.51–1.45; four case-control studies) with low heterogeneity and Eggers *p*-value 0.35.

Maternal exposure to X-ray during pregnancy was not associated with NB risk based on three case-control studies (ES 1.11, CI 0.77–1.59, *p* = 0.65), with no heterogeneity across studies. Likewise, there were no associations observed between paternal occupational exposure to ELF-MF and the risk of NB (Table 2). Maternal residential proximity of <500 m to major roads was elevated (RR 1.23, CI 0.91–1.67, 1 cohort study) when compared to those living ≥500 m away from major roads; but this is based on only one study with a wide confidence interval.

## 4 Discussion

In this systematic review and meta-analysis including 50 epidemiological studies with an approximate total of 14,000 cases of NB. We synthesized the evidence of factors that have been studied in relation to NB in children. Breastfeeding was beneficial with longer duration (≥6 months). Maternal occupational exposure to pesticides during preconception/pregnancy was associated with an increased risk of NB. High birthweight (>4,000 g) showed a slightly elevated ES with borderline significance, as well as for C-section. Associations seen with parental smoking (for paternal in case control studies with self-reported information only) are weak and partly inconsistent, not allowing to draw clear conclusions, but suggesting very modest associations if any. The remaining studied risk factors including gestational age and size, ART and hormonal/infertility treatment, birth order (2 and ≥3), gestational diabetes and pre-eclampsia, maternal alcohol consumption and exposure to X-ray during pregnancy, paternal occupational exposure to pesticides and ELF-MF at the levels studied were not associated with NB risk.

The protective effect of breastfeeding ≥6 months in our study is consistent with other reviews, were the authors reported 39% (28) and 46 % (29) lower risks of NB for longest breastfeeding vs. shortest breastfeeding. Similar findings on the protective effect of breastfeeding ≥6 months have also been reported for other childhood cancer types like leukemia and Wilms tumor (22, 30). The mechanism by which breast milk can reduce the risk of NB is not fully understood. However, breast milk has been reported to contain immunologically active components and multifactorial anti-inflammatory defense mechanisms that influence the development of the immune system of the breastfed infants. Tumor necrosis factor (TNF) -related apoptosis-inducing ligand (TRAIL) in breast milk can control apoptosis and cell proliferation in various organs and tissues (28, 29, 31).

We reported that C-section was suggestive for the risk of NB. Elective C-section is gradually becoming more frequent especially in high socioeconomic status populations, and due to improved surgical procedures in develop countries (32). Systematic reviews and meta-analyses have reported associations between C-sections and childhood leukemia (30, 33) and Wilms tumor (22, 34). C-section has been hypothesized to negatively impact on the function of the developing immune system. The mechanisms for the association between C-section and increased risk of childhood cancer is thought to be due the fact that these neonates do not undergo the essential stress during vaginal delivery that activates the hypothalamic–pituitary–adrenal axis and prime the immune system for future function. Hence creating a permissive environment for malignancies to develop (35–37).

The association we reported for high birthweight (>4,000 g) and increased risk of NB in the present systematic review and meta-analyses was driven by studies published before 2010 and those conducted in North America. Our finding is in agreement with the meta-analysis conducted by Harder et al. (38) who also found an association with slightly lower magnitude (1.19) compared to the present systematic review and meta-analysis (1.21) including subsequent studies, and excluding those with substantial overlaps. Birthweights may affect C-section delivery rates, as small and large new-borns have more C-section deliveries than those of average weight (39). Underlying genetic and epigenetic mechanisms play significant role in high birthweight and childhood cancer. There are several known susceptibility genes, pediatric overgrowth disorders and factors that may influence the association between high birthweight and childhood cancers such as Beckwith–Wiedemann syndrome (BWS), Weaver syndrome, CLOVES, Proteus syndrome, Simpson–Golabi–Behmel syndrome, kaposiform hemangioendothelioma, macrosomia, and organomegaly. About 5%−10% of children with BWS may develop childhood cancer especially Wilms tumor (40–42).

The association we observed for maternal exposure to pesticides during preconception/pregnancy in the main study, was only elevated in sub analysis but with consistent magnitude across decades and in Europe and North America where the studies were conducted. This results are consistent with the findings of Khan et al. (43) who found an association for prenatal pesticides exposure with the same magnitude of association as those reported in the present systematic review and meta-analysis. The mechanisms underlying the associations of pesticides with childhood cancer may differ depending on the type and composition of pesticides. For example, pyrethroids used for pest control on fruits, vegetables as well as for household insecticides, have been reported to induce multiple biological effects (genotoxic and non-genotoxic effects) and initiation the development of childhood cancer (44–46). These insecticides may pass through the feto-placental barrier and thus expose the fetus (47–49). During intrauterine life, there are immunological adaptations to ensure optimal fetal development (50). However, exposure to pesticides induces modifications in the immune system according to the specific pesticide altering the well-regulated immune responses to tumor and microbial antigens, and potentially increasing susceptibility, and development of cancers (51–53). Clear interpretation is hampered by the fact that there are no data on specific active ingredients in pesticides.

### 4.1 Strengths and limitations

This systematic review and meta-analysis was limited by the number of eligible articles which was small for most risk factors, hence, results should be interpreted with caution. Others include potential information and selection biases inherent in the studies, crude exposure assessment methods and exposure misclassification, most likely non-differential, may also have influenced the results. Majority of the studies were conducted in Europe and North America.

Our study also has some strengths, including well-structured search strategy, separation of case-control and cohort/registry-based case-control studies in the meta-analysis. Group of persons exposed (paternal, maternal and childhood) and exposure time window (preconception, prenatal and postnatal) were also separated.

## 5 Conclusion

The present systematic review and meta-analysis suggests that breastfeeding reduces the risk of NB, while maternal occupational pesticides exposure, high birthweight and C-section show a modest association with NB. Improved exposure assessment is needed in further studies including stratification by risk groups, to obtain solid evidence of modifiable risk factors of NB.

## Data Availability

The original contributions presented in the study are included in the article/Supplementary material, further inquiries can be directed to the corresponding author.
