# Supplementary material for: Risk factors of neuroblastoma: a systematic review and meta-analysis
Source: Front Public Health. 2025 Jun 25;13:1576101. doi: 10.3389/fpubh.2025.1576101 (PMC12237980; doi:10.3389/fpubh.2025.1576101)
Supplement: Supplementary file 1 [file Data_Sheet_1.docx]

**Supplementary materials**

**Risk factors of neuroblastoma: a systematic review and meta-analysis**

Felix M. Onyije *, Roya Dolatkhah, Ann Olsson, Liacine Bouaoun, and Joachim Schüz

**Corresponding Author:** *[onyijef@iarc.who.int](mailto:onyijef@iarc.who.int)

Environment and Lifestyle Epidemiology Branch, International Agency for Research on Cancer (IARC/WHO), 25 avenue Tony Garnier, CS 90627, 69366 LYON CEDEX 07, France

**Table of Contents**

Abbreviations: ...........................................................................................................................2

**Appendix 1:** PRISMA checklist, search strategies, and inclusion and exclusion criteria.........3

Table S1: PRISMA 2020 check ……....................................................................................... 3

Table S2: Inclusion and exclusion criteria…….........................................................................6

Table S3: PubMed Search Strategy............................................................................................7

Table S4: Web of Science search strategy...............................................................................12

Table S5: Embase search strategy............................................................................................13

**Appendix 2:** JBI critical appraisal checklist for case-control and cohort studies on NB........14

## Table S6: JBI critical appraisal checklist for case-control studies on NB…………………...14

## Table S7: JBI critical appraisal checklist for cohort studies on NB……………………...….15

**Appendix 3:** Forest and funnel plots of exposures and the risk of NB…………...……...….16

Figure S1-16**:** Forest and funnel plots of birth and parental characteristics…………………16

Figure S17-20: Forest and funnel plots of parental lifestyle and the risk of NB.....................30

Figure S21: Forest and funnel plots of pesticides and the risk of NB.....................................35

Figure S22-24: Forest and funnel plots of parental radiation and the risk of NB....................37

**Reference**: ...............................................................................................................................38

**Abbreviations**

**NB** - Neuroblastoma

**N** - Number of studies

**ES** - Effect size

**LCI** - Lower confidence interval

**UCI** - Upper confidence interval

**I^2^** – Heterogeneity

**Asterisk (*)** - Cohort studies

**Appendix 1: PRISMA check list, search strategies, and inclusion and exclusion criteria**

**eTable 1: PRISMA 2020 check list**

| **Section and Topic** | **Item #** | **Checklist item** | **Location where item is reported** |
| --- | --- | --- | --- |
| **TITLE** | | |  |
| Title | 1 | Identify the report as a systematic review. | **Title** |
| **ABSTRACT** | | |  |
| Abstract | 2 | See the PRISMA 2020 for Abstracts checklist. | **Abstract** |
| **INTRODUCTION** | | |  |
| Rationale | 3 | Describe the rationale for the review in the context of existing knowledge. | **Introduction** |
| Objectives | 4 | Provide an explicit statement of the objective(s) or question(s) the review addresses. | " |
| **METHODS** | | |  |
| Eligibility criteria | 5 | Specify the inclusion and exclusion criteria for the review and how studies were grouped for the syntheses. | **Methods** |
| Information sources | 6 | Specify all databases, registers, websites, organisations, reference lists and other sources searched or consulted to identify studies. Specify the date when each source was last searched or consulted. | " |
| Search strategy | 7 | Present the full search strategies for all databases, registers and websites, including any filters and limits used. | " |
| Selection process | 8 | Specify the methods used to decide whether a study met the inclusion criteria of the review, including how many reviewers screened each record and each report retrieved, whether they worked independently, and if applicable, details of automation tools used in the process. | " |
| Data collection process | 9 | Specify the methods used to collect data from reports, including how many reviewers collected data from each report, whether they worked independently, any processes for obtaining or confirming data from study investigators, and if applicable, details of automation tools used in the process. | " |
| Data items | 10a | List and define all outcomes for which data were sought. Specify whether all results that were compatible with each outcome domain in each study were sought (e.g. for all measures, time points, analyses), and if not, the methods used to decide which results to collect. | " |
|  | 10b | List and define all other variables for which data were sought (e.g. participant and intervention characteristics, funding sources). Describe any assumptions made about any missing or unclear information. | " |
| Study risk of bias assessment | 11 | Specify the methods used to assess risk of bias in the included studies, including details of the tool(s) used, how many reviewers assessed each study and whether they worked independently, and if applicable, details of automation tools used in the process. | " |
| Effect measures | 12 | Specify for each outcome the effect measure(s) (e.g. risk ratio, mean difference) used in the synthesis or presentation of results. | " |
| Synthesis methods | 13a | Describe the processes used to decide which studies were eligible for each synthesis (e.g. tabulating the study intervention characteristics and comparing against the planned groups for each synthesis (item #5)). | " |
|  | 13b | Describe any methods required to prepare the data for presentation or synthesis, such as handling of missing summary statistics, or data conversions. | " |
|  | 13c | Describe any methods used to tabulate or visually display results of individual studies and syntheses. | " |
|  | 13d | Describe any methods used to synthesize results and provide a rationale for the choice(s). If meta-analysis was performed, describe the model(s), method(s) to identify the presence and extent of statistical heterogeneity, and software package(s) used. | " |
|  | 13e | Describe any methods used to explore possible causes of heterogeneity among study results (e.g. subgroup analysis, meta-regression). | " |
|  | 13f | Describe any sensitivity analyses conducted to assess robustness of the synthesized results. | " |
| Reporting bias assessment | 14 | Describe any methods used to assess risk of bias due to missing results in a synthesis (arising from reporting biases). | " |
| Certainty assessment | 15 | Describe any methods used to assess certainty (or confidence) in the body of evidence for an outcome. | " |
| **RESULTS** | | |  |
| Study selection | 16a | Describe the results of the search and selection process, from the number of records identified in the search to the number of studies included in the review, ideally using a flow diagram. | **Results** |
|  | 16b | Cite studies that might appear to meet the inclusion criteria, but which were excluded, and explain why they were excluded. | " |
| Study characteristics | 17 | Cite each included study and present its characteristics. | " |
| Risk of bias in studies | 18 | Present assessments of risk of bias for each included study. | " |
| Results of individual studies | 19 | For all outcomes, present, for each study: (a) summary statistics for each group (where appropriate) and (b) an effect estimates and its precision (e.g. confidence/credible interval), ideally using structured tables or plots. | " |
| Results of syntheses | 20a | For each synthesis, briefly summarise the characteristics and risk of bias among contributing studies. | " |
|  | 20b | Present results of all statistical syntheses conducted. If meta-analysis was done, present for each the summary estimate and its precision (e.g. confidence/credible interval) and measures of statistical heterogeneity. If comparing groups, describe the direction of the effect. | " |
|  | 20c | Present results of all investigations of possible causes of heterogeneity among study results. | " |
|  | 20d | Present results of all sensitivity analyses conducted to assess the robustness of the synthesized results. | " |
| Reporting biases | 21 | Present assessments of risk of bias due to missing results (arising from reporting biases) for each synthesis assessed. | " |
| Certainty of evidence | 22 | Present assessments of certainty (or confidence) in the body of evidence for each outcome assessed. | " |
| **DISCUSSION** | | |  |
| Discussion | 23a | Provide a general interpretation of the results in the context of other evidence. | **Discussion** |
|  | 23b | Discuss any limitations of the evidence included in the review. | " |
|  | 23c | Discuss any limitations of the review processes used. | " |
|  | 23d | Discuss implications of the results for practice, policy, and future research. | " |
| **OTHER INFORMATION** | | |  |
| Registration and protocol | 24a | Provide registration information for the review, including register name and registration number, or state that the review was not registered. | **NIL** |
|  | 24b | Indicate where the review protocol can be accessed, or state that a protocol was not prepared. | **Methods** |
|  | 24c | Describe and explain any amendments to information provided at registration or in the protocol. | **Nil** |
| Support | 25 | Describe sources of financial or non-financial support for the review, and the role of the funders or sponsors in the review. | **Methods** |
| Competing interests | 26 | Declare any competing interests of review authors. | **Page 12** |
| Availability of data, code and other materials | 27 | Report which of the following are publicly available and where they can be found: template data collection forms; data extracted from included studies; data used for all analyses; analytic code; any other materials used in the review. | **Included in the publication** |

*From:*  Page MJ, McKenzie JE, Bossuyt PM, Boutron I, Hoffmann TC, Mulrow CD, et al. The PRISMA 2020 statement: an updated guideline for reporting systematic reviews. BMJ 2021;372:n71.

doi: 10.1136/bmj.n71 For more information, visit: <http://www.prisma-statement.org/>

**eTable 2: Inclusion and exclusion criteria**

|  | **Inclusion Criteria** | **Exclusion Criteria** |
| --- | --- | --- |
| Patient/population | Studies reporting risk estimates for children below age 20 years. | Studies reporting risk estimates for children above age 20 years. |
| Exposure | Childhood or parental exposure to environmental pollutants, chemicals, occupational, or lifestyle (e.g. cigarette smoking), with specific exposure window (preconception, prenatal or postnatal) | Other risk factors including genetic predisposition.  Assessment without exposure window |
| Study design | Case control or cohort studies with risk estimate and 95% CI  Specific exposure window e.g. preconception, prenatal or postnatal | Ecological and cross-sectional studies  Pooled analysis  Studies without risk estimates or 95% CI  Assessment without exposure window |
| Outcome | Neuroblastoma | Other types childhood cancers  Non-specified type of cancer (combination of different cancer types or sites) |
| Search Criteria | No language or publication date restrictions |  |

**eTable 3: PubMed Search Strategy**

| **Search No** | **Query** | | **Search Details** | **Results** |
| --- | --- | --- | --- | --- |
| 1 | "Child*" OR "infan*" OR "New-born" OR "pediatric" OR "paediatric" OR "Childhood" OR "Adolescence" OR "Teenage*" OR "Youth*" | | "child*"[All Fields] OR "infan*"[All Fields] OR "New-born"[All Fields] OR "pediatric"[All Fields] OR "paediatric"[All Fields] OR "Childhood"[All Fields] OR "Adolescence"[All Fields] OR "teenage*"[All Fields] OR "youth*"[All Fields] | **3,824,000** |
| 2 | "Environmental Pollution"[Mesh] OR "Environmental Exposure" OR "Occupational Exposure"[Mesh]OR "Occupational Exposure" OR "Prenatal exposure" OR "Maternal exposure" OR "Paternal exposure" OR "Residential exposure" OR "household exposure" OR "domestic exposure" OR "Indoor exposure" OR "Outdoor exposure" OR "Smok*" OR "Tobacco" OR "Cigarette" OR, "Radiation*" OR "birth weight" OR "Chemical exposure" OR "lifestyle" OR "electromagnetic fields"[MeSH Terms] OR "non-ionising radiation" OR "radiation" OR "phototherapy" OR "Breast feeding" OR "Birth characteristics" OR "Electromagnetic fields" OR "Alcohol" OR "Folic acid" OR "Vitamins" OR "Cesarean Section" OR "Smoking" OR "Paint" OR "Coffee" OR "Tea" OR "Livestock" OR "Birth weight" OR "Parity" OR "Birth order" OR "Gestation" OR "Age" OR "ART" OR "Pesticides" | | (("Environmental Pollution"[MeSH Terms] OR "Environmental Exposure"[All Fields] OR "Occupational Exposure"[MeSH Terms] OR "Occupational Exposure"[All Fields] OR "Prenatal exposure"[All Fields] OR "Maternal exposure"[All Fields] OR "Paternal exposure"[All Fields] OR "Residential exposure"[All Fields] OR "household exposure"[All Fields] OR "domestic exposure"[All Fields] OR "Indoor exposure"[All Fields] OR "Outdoor exposure"[All Fields] OR "smok*"[All Fields] OR "Tobacco"[All Fields] OR "cigarette ("[All Fields])) AND "radiation*"[All Fields]) OR "Birth weight"[All Fields] OR "Chemical exposure"[All Fields] OR "lifestyle"[All Fields] OR "Electromagnetic fields"[MeSH Terms] OR "non-ionising radiation"[All Fields] OR "radiation"[All Fields] OR "phototherapy"[All Fields] OR "Breast feeding"[All Fields] OR "Birth characteristics"[All Fields] OR "Electromagnetic fields"[All Fields] OR "Alcohol"[All Fields] OR "Folic acid"[All Fields] OR "Vitamins"[All Fields] OR "Cesarean Section"[All Fields] OR "Smoking"[All Fields] OR "Paint"[All Fields] OR "Coffee"[All Fields] OR "Tea"[All Fields] OR "Livestock"[All Fields] OR "Birth weight"[All Fields] OR "Parity"[All Fields] OR "Birth order"[All Fields] OR "Gestation"[All Fields] OR "Age"[All Fields] OR "ART"[All Fields] OR "Pesticides"[All Fields] | **8,730,149** |
| 3 | "Case-Control Stud*"[Mesh] OR "Case-Control Stud*" OR "cohort" OR "Epidemiology"[SH] "observational study" | | ("case control stud*"[MeSH Terms] OR "case control stud*"[All Fields] OR "cohort"[All Fields] OR "Epidemiology"[MeSH Subheading]) AND "observational study"[All Fields] | **118,206** |
| 4 | "Brain" OR "spinal cord" OR "Neuroblastoma" OR "Wilms tumor" OR "Wilms tumour" OR "Lymphoma" OR "Hodgkin lymphoma" OR "non-Hodgkin lymphoma" OR "Germ cell" OR "Soft tissue" OR "Rhabdomyosarcoma" OR "Retinoblastoma" OR "osteosarcoma" OR "sarcoma" OR "Hepatoblastoma" "Neoplasms"[Mesh] OR "cancer*" OR "cancerous" OR "carcinoma" OR "neoplasm*" OR "tumor*" OR "tumour*" OR "malignan*" | | (("Brain"[All Fields] OR "spinal cord"[All Fields] OR "Neuroblastoma"[All Fields] OR "Wilms tumor"[All Fields] OR "Wilms tumour"[All Fields] OR "Lymphoma"[All Fields] OR "Hodgkin lymphoma"[All Fields] OR "non-hodgkin lymphoma"[All Fields] OR "Germ cell"[All Fields] OR "Soft tissue"[All Fields] OR "Rhabdomyosarcoma"[All Fields] OR "Retinoblastoma"[All Fields] OR "osteosarcoma"[All Fields] OR "sarcoma"[All Fields] OR "Hepatoblastoma"[All Fields]) AND "Neoplasms"[MeSH Terms]) OR "cancer*"[All Fields] OR "cancerous"[All Fields] OR "carcinoma"[All Fields] OR "neoplasm*"[All Fields] OR "tumor*"[All Fields] OR "tumour*"[All Fields] OR "malignan*"[All Fields] | **5,032,012** |
| 5 | #1 AND #2 AND #3 AND #4 | | ("child*"[All Fields] OR "infan*"[All Fields] OR "New-born"[All Fields] OR "pediatric"[All Fields] OR "paediatric"[All Fields] OR "Childhood"[All Fields] OR "Adolescence"[All Fields] OR "teenage*"[All Fields] OR "youth*"[All Fields]) AND ((("Environmental Pollution"[MeSH Terms] OR "Environmental Exposure"[All Fields] OR "Occupational Exposure"[MeSH Terms] OR "Occupational Exposure"[All Fields] OR "Prenatal exposure"[All Fields] OR "Maternal exposure"[All Fields] OR "Paternal exposure"[All Fields] OR "Residential exposure"[All Fields] OR "household exposure"[All Fields] OR "domestic exposure"[All Fields] OR "Indoor exposure"[All Fields] OR "Outdoor exposure"[All Fields] OR "smok*"[All Fields] OR "Tobacco"[All Fields] OR "cigarette ("[All Fields])) AND "radiation*"[All Fields]) OR "Birth weight"[All Fields] OR "Chemical exposure"[All Fields] OR "lifestyle"[All Fields] OR "Electromagnetic fields"[MeSH Terms] OR "non-ionising radiation"[All Fields] OR "radiation"[All Fields] OR "phototherapy"[All Fields] OR "Breast feeding"[All Fields] OR "Birth characteristics"[All Fields] OR "Electromagnetic fields"[All Fields] OR "Alcohol"[All Fields] OR "Folic acid"[All Fields] OR "Vitamins"[All Fields] OR "Cesarean Section"[All Fields] OR "Smoking"[All Fields] OR "Paint"[All Fields] OR "Coffee"[All Fields] OR "Tea"[All Fields] OR "Livestock"[All Fields] OR "Birth weight"[All Fields] OR "Parity"[All Fields] OR "Birth order"[All Fields] OR "Gestation"[All Fields] OR "Age"[All Fields] OR "ART"[All Fields] OR "Pesticides"[All Fields]) AND (("case control stud*"[MeSH Terms] OR "case control stud*"[All Fields] OR "cohort"[All Fields] OR "Epidemiology"[MeSH Subheading]) AND "observational study"[All Fields]) AND ((("Brain"[All Fields] OR "spinal cord"[All Fields] OR "Neuroblastoma"[All Fields] OR "Wilms tumor"[All Fields] OR "Wilms tumour"[All Fields] OR "Lymphoma"[All Fields] OR "Hodgkin lymphoma"[All Fields] OR "non-hodgkin lymphoma"[All Fields] OR "Germ cell"[All Fields] OR "Soft tissue"[All Fields] OR "Rhabdomyosarcoma"[All Fields] OR "Retinoblastoma"[All Fields] OR "osteosarcoma"[All Fields] OR "sarcoma"[All Fields] OR "Hepatoblastoma"[All Fields]) AND "Neoplasms"[MeSH Terms]) OR "cancer*"[All Fields] OR "cancerous"[All Fields] OR "carcinoma"[All Fields] OR "neoplasm*"[All Fields] OR "tumor*"[All Fields] OR "tumour*"[All Fields] OR "malignan*"[All Fields]) | **1,469** |
|  |  | **Filters** |  |  |
| 6 | #1 AND #2 AND #3 AND #4 | Abstract | (("child*"[All Fields] OR "infan*"[All Fields] OR "New-born"[All Fields] OR "pediatric"[All Fields] OR "paediatric"[All Fields] OR "Childhood"[All Fields] OR "Adolescence"[All Fields] OR "teenage*"[All Fields] OR "youth*"[All Fields]) AND ((("Environmental Pollution"[MeSH Terms] OR "Environmental Exposure"[All Fields] OR "Occupational Exposure"[MeSH Terms] OR "Occupational Exposure"[All Fields] OR "Prenatal exposure"[All Fields] OR "Maternal exposure"[All Fields] OR "Paternal exposure"[All Fields] OR "Residential exposure"[All Fields] OR "household exposure"[All Fields] OR "domestic exposure"[All Fields] OR "Indoor exposure"[All Fields] OR "Outdoor exposure"[All Fields] OR "smok*"[All Fields] OR "Tobacco"[All Fields] OR "cigarette ("[All Fields])) AND "radiation*"[All Fields]) OR "Birth weight"[All Fields] OR "Chemical exposure"[All Fields] OR "lifestyle"[All Fields] OR "Electromagnetic fields"[MeSH Terms] OR "non-ionising radiation"[All Fields] OR "radiation"[All Fields] OR "phototherapy"[All Fields] OR "Breast feeding"[All Fields] OR "Birth characteristics"[All Fields] OR "Electromagnetic fields"[All Fields] OR "Alcohol"[All Fields] OR "Folic acid"[All Fields] OR "Vitamins"[All Fields] OR "Cesarean Section"[All Fields] OR "Smoking"[All Fields] OR "Paint"[All Fields] OR "Coffee"[All Fields] OR "Tea"[All Fields] OR "Livestock"[All Fields] OR "Birth weight"[All Fields] OR "Parity"[All Fields] OR "Birth order"[All Fields] OR "Gestation"[All Fields] OR "Age"[All Fields] OR "ART"[All Fields] OR "Pesticides"[All Fields]) AND (("case control stud*"[MeSH Terms] OR "case control stud*"[All Fields] OR "cohort"[All Fields] OR "Epidemiology"[MeSH Subheading]) AND "observational study"[All Fields]) AND ((("Brain"[All Fields] OR "spinal cord"[All Fields] OR "Neuroblastoma"[All Fields] OR "Wilms tumor"[All Fields] OR "Wilms tumour"[All Fields] OR "Lymphoma"[All Fields] OR "Hodgkin lymphoma"[All Fields] OR "non-hodgkin lymphoma"[All Fields] OR "Germ cell"[All Fields] OR "Soft tissue"[All Fields] OR "Rhabdomyosarcoma"[All Fields] OR "Retinoblastoma"[All Fields] OR "osteosarcoma"[All Fields] OR "sarcoma"[All Fields] OR "Hepatoblastoma"[All Fields]) AND "Neoplasms"[MeSH Terms]) OR "cancer*"[All Fields] OR "cancerous"[All Fields] OR "carcinoma"[All Fields] OR "neoplasm*"[All Fields] OR "tumor*"[All Fields] OR "tumour*"[All Fields] OR "malignan*"[All Fields])) AND (fha[Filter]) | **1,459** |
| 7 | #1 AND #2 AND #3 AND #4 | Abstract, Humans | (("child*"[All Fields] OR "infan*"[All Fields] OR "New-born"[All Fields] OR "pediatric"[All Fields] OR "paediatric"[All Fields] OR "Childhood"[All Fields] OR "Adolescence"[All Fields] OR "teenage*"[All Fields] OR "youth*"[All Fields]) AND ((("Environmental Pollution"[MeSH Terms] OR "Environmental Exposure"[All Fields] OR "Occupational Exposure"[MeSH Terms] OR "Occupational Exposure"[All Fields] OR "Prenatal exposure"[All Fields] OR "Maternal exposure"[All Fields] OR "Paternal exposure"[All Fields] OR "Residential exposure"[All Fields] OR "household exposure"[All Fields] OR "domestic exposure"[All Fields] OR "Indoor exposure"[All Fields] OR "Outdoor exposure"[All Fields] OR "smok*"[All Fields] OR "Tobacco"[All Fields] OR "cigarette ("[All Fields])) AND "radiation*"[All Fields]) OR "Birth weight"[All Fields] OR "Chemical exposure"[All Fields] OR "lifestyle"[All Fields] OR "Electromagnetic fields"[MeSH Terms] OR "non-ionising radiation"[All Fields] OR "radiation"[All Fields] OR "phototherapy"[All Fields] OR "Breast feeding"[All Fields] OR "Birth characteristics"[All Fields] OR "Electromagnetic fields"[All Fields] OR "Alcohol"[All Fields] OR "Folic acid"[All Fields] OR "Vitamins"[All Fields] OR "Cesarean Section"[All Fields] OR "Smoking"[All Fields] OR "Paint"[All Fields] OR "Coffee"[All Fields] OR "Tea"[All Fields] OR "Livestock"[All Fields] OR "Birth weight"[All Fields] OR "Parity"[All Fields] OR "Birth order"[All Fields] OR "Gestation"[All Fields] OR "Age"[All Fields] OR "ART"[All Fields] OR "Pesticides"[All Fields]) AND (("case control stud*"[MeSH Terms] OR "case control stud*"[All Fields] OR "cohort"[All Fields] OR "Epidemiology"[MeSH Subheading]) AND "observational study"[All Fields]) AND ((("Brain"[All Fields] OR "spinal cord"[All Fields] OR "Neuroblastoma"[All Fields] OR "Wilms tumor"[All Fields] OR "Wilms tumour"[All Fields] OR "Lymphoma"[All Fields] OR "Hodgkin lymphoma"[All Fields] OR "non-hodgkin lymphoma"[All Fields] OR "Germ cell"[All Fields] OR "Soft tissue"[All Fields] OR "Rhabdomyosarcoma"[All Fields] OR "Retinoblastoma"[All Fields] OR "osteosarcoma"[All Fields] OR "sarcoma"[All Fields] OR "Hepatoblastoma"[All Fields]) AND "Neoplasms"[MeSH Terms]) OR "cancer*"[All Fields] OR "cancerous"[All Fields] OR "carcinoma"[All Fields] OR "neoplasm*"[All Fields] OR "tumor*"[All Fields] OR "tumour*"[All Fields] OR "malignan*"[All Fields])) AND ((fha[Filter]) AND (humans[Filter])) | **1,416** |
| 8 | #1 AND #2 AND #3 AND #4 | Abstract, Humans,  Child: birth-18 years | (("child*"[All Fields] OR "infan*"[All Fields] OR "New-born"[All Fields] OR "pediatric"[All Fields] OR "paediatric"[All Fields] OR "Childhood"[All Fields] OR "Adolescence"[All Fields] OR "teenage*"[All Fields] OR "youth*"[All Fields]) AND ((("Environmental Pollution"[MeSH Terms] OR "Environmental Exposure"[All Fields] OR "Occupational Exposure"[MeSH Terms] OR "Occupational Exposure"[All Fields] OR "Prenatal exposure"[All Fields] OR "Maternal exposure"[All Fields] OR "Paternal exposure"[All Fields] OR "Residential exposure"[All Fields] OR "household exposure"[All Fields] OR "domestic exposure"[All Fields] OR "Indoor exposure"[All Fields] OR "Outdoor exposure"[All Fields] OR "smok*"[All Fields] OR "Tobacco"[All Fields] OR "cigarette ("[All Fields])) AND "radiation*"[All Fields]) OR "Birth weight"[All Fields] OR "Chemical exposure"[All Fields] OR "lifestyle"[All Fields] OR "Electromagnetic fields"[MeSH Terms] OR "non-ionising radiation"[All Fields] OR "radiation"[All Fields] OR "phototherapy"[All Fields] OR "Breast feeding"[All Fields] OR "Birth characteristics"[All Fields] OR "Electromagnetic fields"[All Fields] OR "Alcohol"[All Fields] OR "Folic acid"[All Fields] OR "Vitamins"[All Fields] OR "Cesarean Section"[All Fields] OR "Smoking"[All Fields] OR "Paint"[All Fields] OR "Coffee"[All Fields] OR "Tea"[All Fields] OR "Livestock"[All Fields] OR "Birth weight"[All Fields] OR "Parity"[All Fields] OR "Birth order"[All Fields] OR "Gestation"[All Fields] OR "Age"[All Fields] OR "ART"[All Fields] OR "Pesticides"[All Fields]) AND (("case control stud*"[MeSH Terms] OR "case control stud*"[All Fields] OR "cohort"[All Fields] OR "Epidemiology"[MeSH Subheading]) AND "observational study"[All Fields]) AND ((("Brain"[All Fields] OR "spinal cord"[All Fields] OR "Neuroblastoma"[All Fields] OR "Wilms tumor"[All Fields] OR "Wilms tumour"[All Fields] OR "Lymphoma"[All Fields] OR "Hodgkin lymphoma"[All Fields] OR "non-hodgkin lymphoma"[All Fields] OR "Germ cell"[All Fields] OR "Soft tissue"[All Fields] OR "Rhabdomyosarcoma"[All Fields] OR "Retinoblastoma"[All Fields] OR "osteosarcoma"[All Fields] OR "sarcoma"[All Fields] OR "Hepatoblastoma"[All Fields]) AND "Neoplasms"[MeSH Terms]) OR "cancer*"[All Fields] OR "cancerous"[All Fields] OR "carcinoma"[All Fields] OR "neoplasm*"[All Fields] OR "tumor*"[All Fields] OR "tumour*"[All Fields] OR "malignan*"[All Fields])) AND ((fha[Filter]) AND (humans[Filter]) AND (allchild[Filter])) | **1,048** |

**eTable 4: Web of Science search strategy**

| **Search No** | **Query** | **Results** |
| --- | --- | --- |
| 1 | **ALL=(“Child*” OR “infan*” OR “New-born” OR “pediatric” OR “paediatric” OR “Childhood” OR “Adolescence” OR “Teenage*” OR “Youth*”)** | **4,195,704** |
| 2 | **TI=(“Environmental Pollution" OR "Environmental Exposure" OR "Occupational Exposure" OR "Occupational Exposure" OR “Prenatal exposure” OR “Maternal exposure”** OR "Paternal exposure"[All Fields] **OR “Residential exposure” OR “household exposure” OR “domestic exposure” OR “Indoor exposure” OR “Outdoor exposure” OR “Smok*” OR “Tobacco” OR “Cigarette” OR, “Radiation*” OR “birth weight” OR “Chemical exposure” OR “lifestyle” OR "electromagnetic fields" OR "non-ionising radiation" OR "radiation” OR “phototherapy” OR “Breast feeding” OR “Birth characteristics” OR “Electromagnetic fields” OR “Alcohol” OR “Folic acid” OR “Vitamins” OR “Cesarean Section” OR “Smoking” OR “Paint” OR “Coffee” OR “Tea” OR “Livestock” OR “Birth weight” OR “Parity” OR “Birth order” OR “Gestation” OR “Age” OR “ART” OR “Pesticides”)** | **1,574,892** |
| 3 | **ALL= ("Case-Control Stud*" OR "Case-Control Stud*” OR “cohort” OR "Epidemiology" OR "observational study")** | **1,917,679** |
| 4 | **TI=(“Brain” OR “spinal cord” OR “Neuroblastoma” OR “Wilms tumor” OR “Wilms tumour” OR “Lymphoma” OR “Hodgkin lymphoma” OR “non-Hodgkin lymphoma” OR “Rhabdomyosarcoma” OR “Retinoblastoma” OR “osteosarcoma” OR “sarcoma” OR “Hepatoblastoma” "Neoplasms" OR “cancer*” OR “cancerous” OR “carcinoma” OR “neoplasm*” OR “tumor*” OR “tumour*” OR “malignan*” )** | **3,389,200** |
| 5 | **#1 AND #2 AND #3 AND #4** | **1,837** |

**eTable 5:** **Embase search strategy**

| **Search No** | **Query** | **Results** |
| --- | --- | --- |
| #1 | ('child*' OR 'infan*' OR 'new-born' OR 'pediatric'/mj OR 'paediatric'/mj OR 'childhood'/mj OR 'adolescence'/mj OR 'teenage*' OR 'youth*':kw) AND [article]/lim AND ([child]/lim OR [adolescent]/lim) AND [humans]/lim AND [abstracts]/lim AND [embase]/lim | **993,352** |
| #2 | ('environmental exposure'/mj OR 'occupational exposure'/mj OR 'prenatal exposure'/mj OR 'maternal exposure'/mj OR 'paternal exposure'/mj OR 'residential exposure'/mj OR 'household exposure' OR 'domestic exposure' OR 'indoor exposure' OR 'outdoor exposure' OR 'tobacco'/mj OR 'cigarette'/mj OR 'chemical' OR 'lifestyle'/mj OR 'non ionising radiation' OR 'radiation'/mj OR 'phototherapy'/mj OR 'breast feeding'/mj OR 'birth characteristics' OR 'electromagnetic fields'/mj OR 'alcohol'/mj OR 'folic acid'/mj OR 'vitamins'/mj OR 'cesarean section'/mj OR 'smoking'/mj OR 'paint'/mj OR 'coffee'/mj OR 'tea'/mj OR 'livestock'/mj OR 'birth weight'/mj OR 'parity'/mj OR 'birth order'/mj OR 'gestation'/mj OR 'age'/mj OR 'art'/mj OR 'pesticides':kw) AND [article]/lim AND ([child]/lim OR [adolescent]/lim) AND [humans]/lim AND [abstracts]/lim AND [embase]/lim | **39,293** |
| #3 | ('case-control stud*' OR 'cohort' OR  'epidemiology'/mj OR 'observational study':kw) AND [article]/lim AND ([child]/lim OR [adolescent]/lim) AND [humans]/lim AND [abstracts]/lim AND [embase]/lim |  |
| #4 | (('brain'/mj OR 'spinal cord'/mj OR 'neuroblastoma'/mj OR 'wilms tumor'/mj OR 'wilms tumour'/mj OR 'lymphoma'/mj OR 'hodgkin lymphoma'/mj OR 'non-hodgkin lymphoma'/mj OR 'germ cell'/mj OR 'rhabdomyosarcoma'/mj OR 'retinoblastoma'/mj OR 'osteosarcoma'/mj OR 'sarcoma'/mj OR 'hepatoblastoma'/mj) AND 'neoplasms'/mj OR 'cancer*' OR 'cancerous' OR 'carcinoma'/mj OR 'neoplasm*' OR 'tumor*' OR 'tumour*' OR 'malignan*':kw) AND [article]/lim AND ([child]/lim OR [adolescent]/lim) AND [humans]/lim AND [abstracts]/lim AND [embase]/lim | **187,915** |
| #5 | #1 AND #2 AND #3 AND #4 | **1,069** |

##

**Appendix 2:** **JBI critical appraisal checklist for case-control and cohort studies on NB**

## **eTable 6:** **JBI critical appraisal checklist for case-control studies on NB**

| **First author, year [reference]** | Free of selection bias? | Appropriate sampling? | The same criteria used for cases and control? | Standard, valid and reliable exposure measurement? | Exposure measured the same way for cases and control? | Identified confounders? | Confounder management strategies stated? | Valid & reliable outcome measure? | Sufficient exposure time? | Appropriate statistical analysis? | Overall |
| --- | --- | --- | --- | --- | --- | --- | --- | --- | --- | --- | --- |
| Schüz et al., 1999 | Y | Y | Y | Y | Y | Y | Y | Y | U | Y | 9 |
| Hardell and Dreifaldt, 2001 | Y | Y | Y | Y | Y | Y | Y | Y | U | Y | 9 |
| Schüz et al., 2001 | Y | Y | Y | Y | Y | Y | Y | Y | U | Y | 9 |
| Pang et al., 2003 | Y | Y | Y | Y | Y | Y | Y | Y | U | Y | 9 |
| Bunin et al., 1990 | Y | Y | Y | Y | Y | Y | Y | Y | U | Y | 9 |
| Schüz et al and Forman, 2007 | Y | Y | Y | Y | Y | Y | Y | Y | U | Y | 9 |
| Schuz et al., 2007 | Y | Y | Y | Y | Y | Y | Y | Y | U | Y | 9 |
| Hug et al., 2010 | Y | Y | Y | Y | Y | Y | Y | Y | U | Y | 9 |
| Rajaraman et al., 2011 | Y | Y | Y | Y | Y | Y | Y | Y | U | Y | 9 |
| Olshan et al. 1999 | Y | Y | Y | Y | Y | Y | Y | Y | U | Y | 9 |
| Olshan et al., 2002 | Y | Y | Y | Y | Y | Y | Y | Y | U | Y | 9 |
| Daniels et al., 2001 | Y | Y | Y | Y | Y | Y | Y | Y | U | Y | 9 |
| Daniels et al., 2002 | Y | Y | Y | Y | Y | Y | Y | Y | U | Y | 9 |
| Rios et al., 2017 | Y | Y | Y | Y | Y | Y | Y | Y | U | Y | 9 |
| Rios et al., 2019 | Y | Y | Y | Y | Y | Y | Y | Y | U | Y | 9 |
| Carozza et al., 2009 | Y | Y | Y | U | Y | Y | Y | Y | U | Y | 8 |
| Munzer et al., 2008 | Y | Y | Y | Y | Y | Y | Y | Y | U | Y | 9 |
| Parodi et al., 2014 | Y | Y | Y | Y | Y | Y | Y | Y | U | Y | 9 |
| Yang et al., 2000 | Y | Y | Y | Y | Y | Y | Y | Y | U | Y | 9 |
| Buck et al., 2001 | Y | Y | Y | Y | Y | Y | Y | Y | U | Y | 9 |
| Hamrick et al., 2001 | Y | Y | Y | Y | Y | Y | Y | Y | U | Y | 9 |
| Patton et al., 2004 | Y | Y | Y | Y | Y | Y | Y | Y | U | Y | 9 |
| De Roos et al., 2001 | Y | Y | Y | Y | Y | Y | Y | Y | U | Y | 9 |
| Kerr et al., 2000 | Y | Y | Y | Y | Y | Y | Y | Y | U | Y | 9 |
| Schwartzbaum, 1992 | Y | U | U | Y | Y | Y | Y | Y | U | Y | 7 |
| **Total (%)** |  |  |  |  |  |  | **222/250*100** | | | | **88.9%** |

Yes=1, No=0, Unclear =0

## **eTable 7: JBI quality appraisal checklist for cohort studies on NB**

| **First author, year** | Free of selection bias? | Clear exposure measurement? | Valid & reliable exposure measurement? | Identified confounders? | Confounder management strategies stated? | Free of outcome sample? | Valid & reliable outcome measure? | Sufficient follow-up time? | Follow-up complete? | Incomplete follow-up strategies? | Appropriate statistical analysis? | Overall |
| --- | --- | --- | --- | --- | --- | --- | --- | --- | --- | --- | --- | --- |
| Feychting et al., 2000 | Y | Y | Y | Y | Y | U | Y | Y | Y | U | Y | 9 |
| Stavrou et al., 2009 | Y | Y | Y | Y | Y | U | Y | U | N | N | Y | 7 |
| Sundh et al., 2014 | Y | Y | Y | Y | Y | U | Y | Y | Y | U | Y | 9 |
| Schüz et al., 2015 | Y | Y | Y | Y | Y | U | Y | Y | Y | U | Y | 9 |
| Mortensen et al., 2016 | Y | Y | Y | Y | Y | U | Y | Y | Y | U | Y | 9 |
| Spector et al., 2019 | Y | Y | Y | Y | Y | U | Y | Y | Y | U | Y | 9 |
| Heck et al., 2020 | Y | Y | Y | Y | Y | U | Y | N | N | U | Y | 7 |
| Bjørge et al., 2008 | Y | Y | Y | Y | Y | U | Y | Y | Y | U | Y | 9 |
|  |  |  |  |  |  | **68/88*100** | | | | | | **77.2%** |
| **Registry-based case-control studies classified as cohort studies** | | | | | | | | | | | | |
| **First author, year [reference]** | Free of selection bias? | Appropriate sampling? | The same criteria used for cases and control? | Standard, valid and reliable exposure measurement? | Exposure measured the same way for cases and control? | Identified confounders? | Confounder management strategies stated? | | Valid & reliable outcome measure? | Sufficient exposure time? | Appropriate statistical analysis? | Overall |
| Contreras et al., 2016 | Y | Y | Y | Y | Y | Y | Y | | Y | NA | Y | 9 |
| Heck et al., 2016 | Y | Y | Y | Y | Y | Y | Y | | Y | U | Y | 9 |
| Contreras et al., 2017 | Y | Y | Y | Y | Y | Y | Y | | Y | U | Y | 9 |
| Seppälä et al., 2021 | Y | Y | Y | Y | Y | Y | Y | | Y | U | Y | 9 |
| Volk et al., 2020 | Y | Y | Y | Y | Y | Y | N | | Y | U | Y | 8 |
| Williams et al., 2021 | Y | Y | Y | Y | Y | Y | Y | | Y | NA | Y | 9 |
| Huang et al., 2022 | Y | Y | Y | Y | Y | Y | Y | | Y | U | Y | 9 |
| Kumar et al., 2018 | Y | Y | Y | Y | Y | Y | Y | | Y | U | Y | 9 |
| Bluhm et al., 2008 | Y | Y | Y | Y | Y | Y | Y | | Y | U | Y | 9 |
| McLaughlin et al., 2009 | Y | Y | Y | Y | Y | Y | Y | | Y | U | Y | 9 |
| Urayama et al., 2007 | Y | Y | Y | Y | Y | Y | Y | | Y | U | Y | 9 |
| Johnson et al., 2008 | Y | Y | Y | Y | Y | Y | Y | | Y | U | Y | 9 |
| Chow et al., 2007 | Y | Y | Y | Y | Y | Y | Y | | Y | U | Y | 9 |
| Johnson and Spitz, 1985 | Y | N | Y | Y | Y | Y | N | | Y | U | Y | 7 |
| Spitz and Johnson, 1985 | Y | N | Y | Y | Y | Y | N | | Y | U | Y | 7 |
| Schraw et al., 2022 | Y | Y | Y | Y | Y | Y | Y | | Y | U | Y | 9 |
| Neglia et al., 1988 | Y | Y | Y | Y | Y | Y | N | | Y | U | Y | 8 |
|  |  |  |  |  |  |  | **147/170*100** | | | | | **86.4%**  **81.8%** |
| **Total percentage** |  |  |  |  |  |  | **77.2+86.4 / 2** | | | | |  |

Yes=1, No=0, Unclear =0

**Appendix 3: Figure S1-16: Forest and funnel plots of birth and parental characteristics**


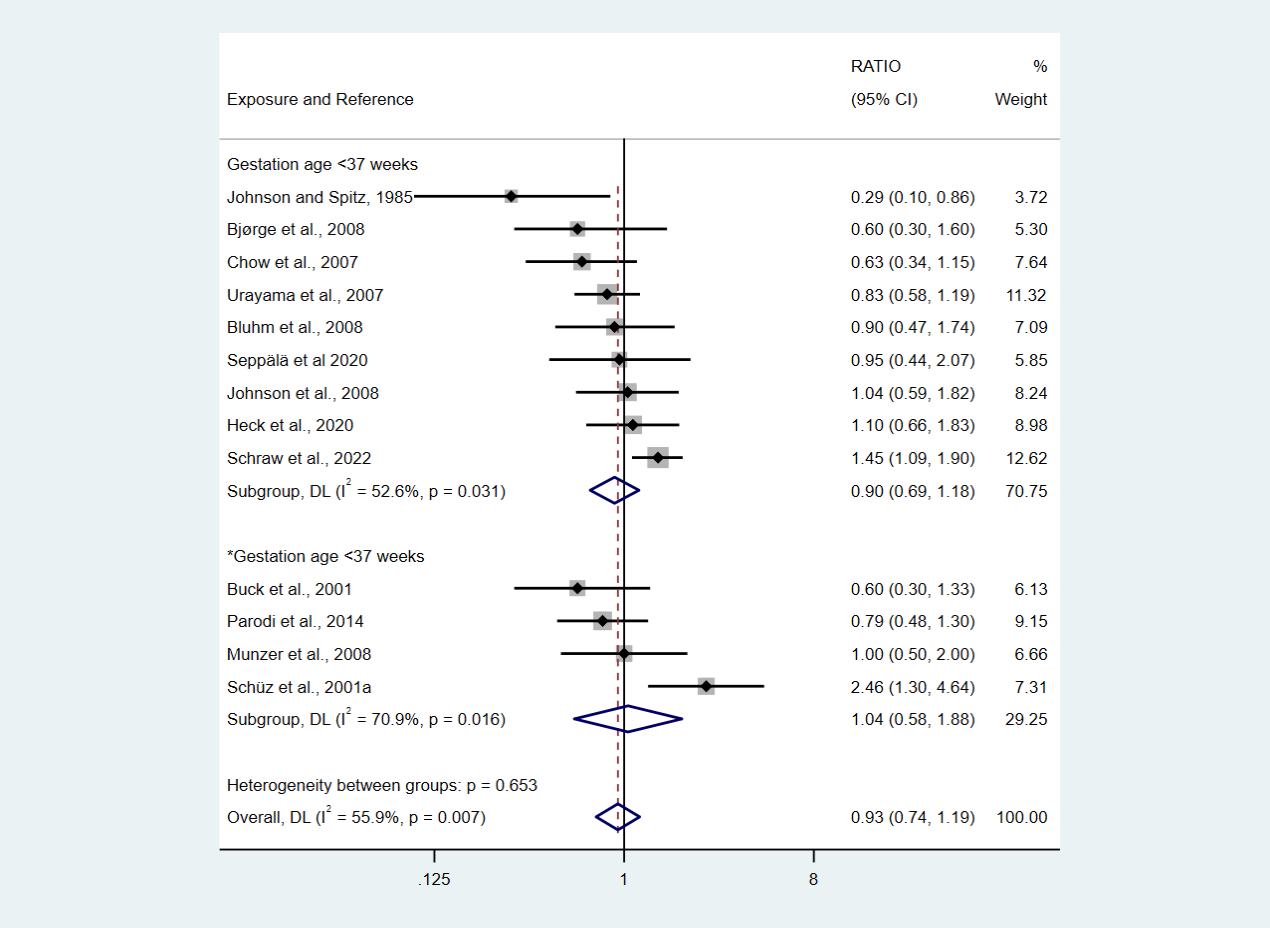


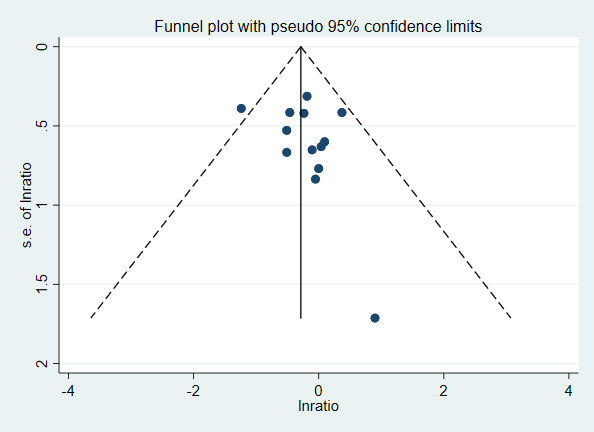


Figure S1: Forest and funnel plots of gestational age < 37 weeks


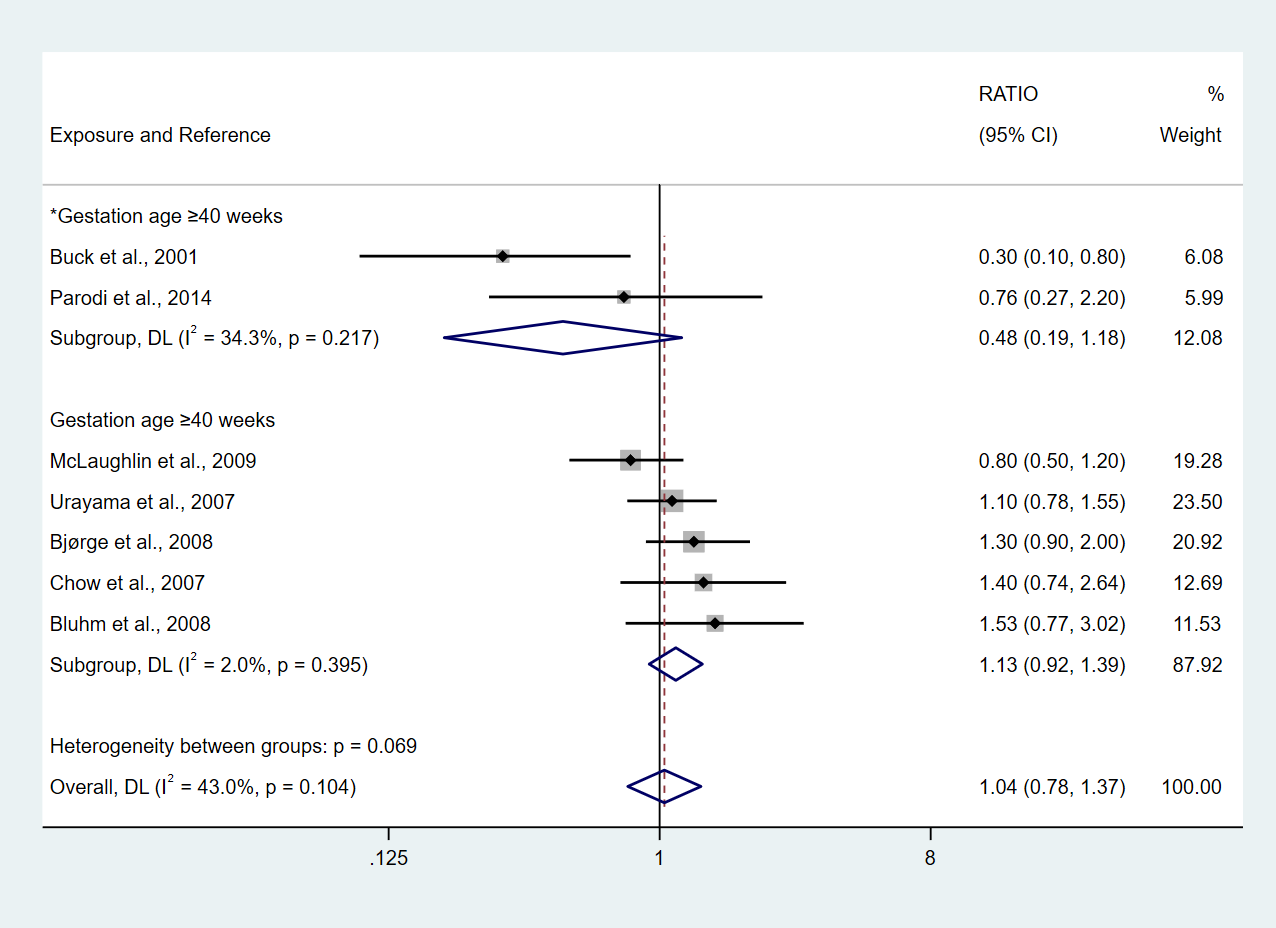


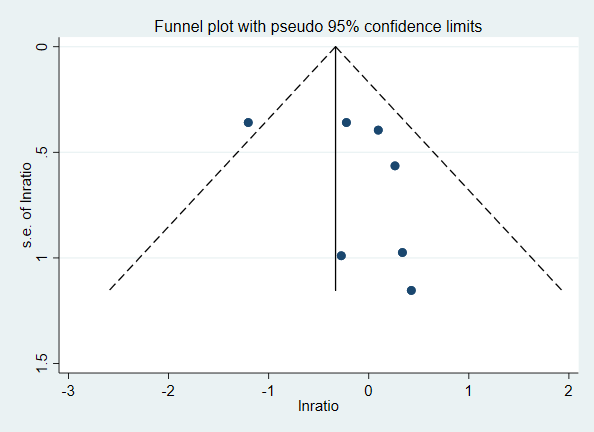


Figure S2: Forest and funnel plots of gestational age >40 weeks


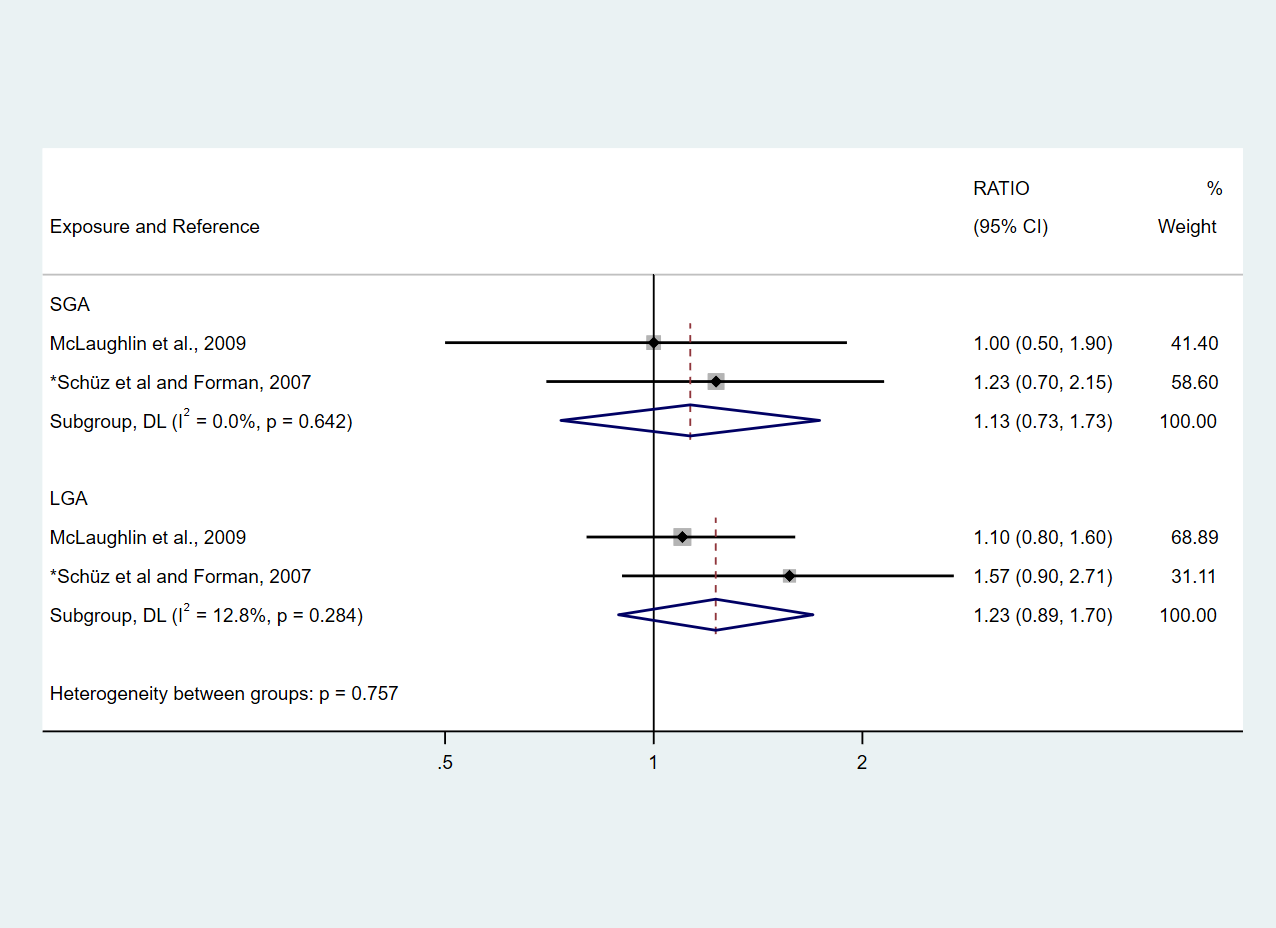


Figure S3: Forest plot of SGA and LGA


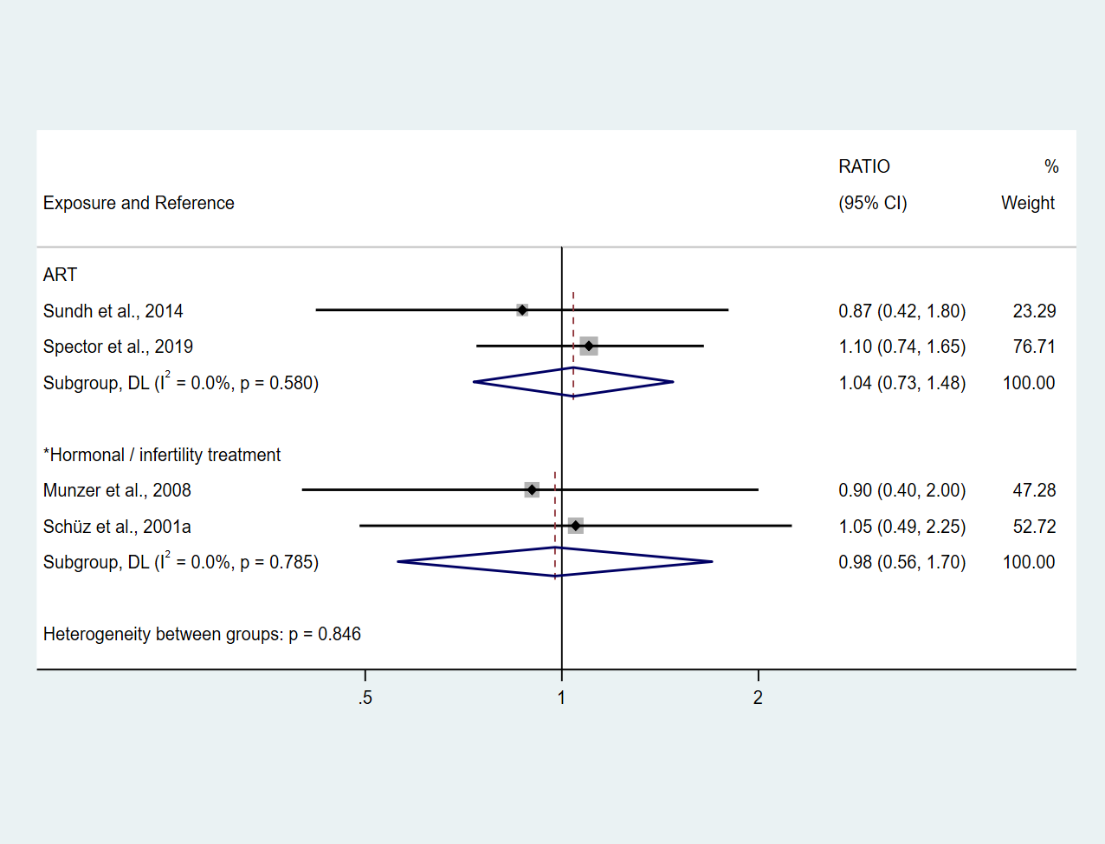


Figure S4: Forest plot of ART and Hormonal/Infertility treatment


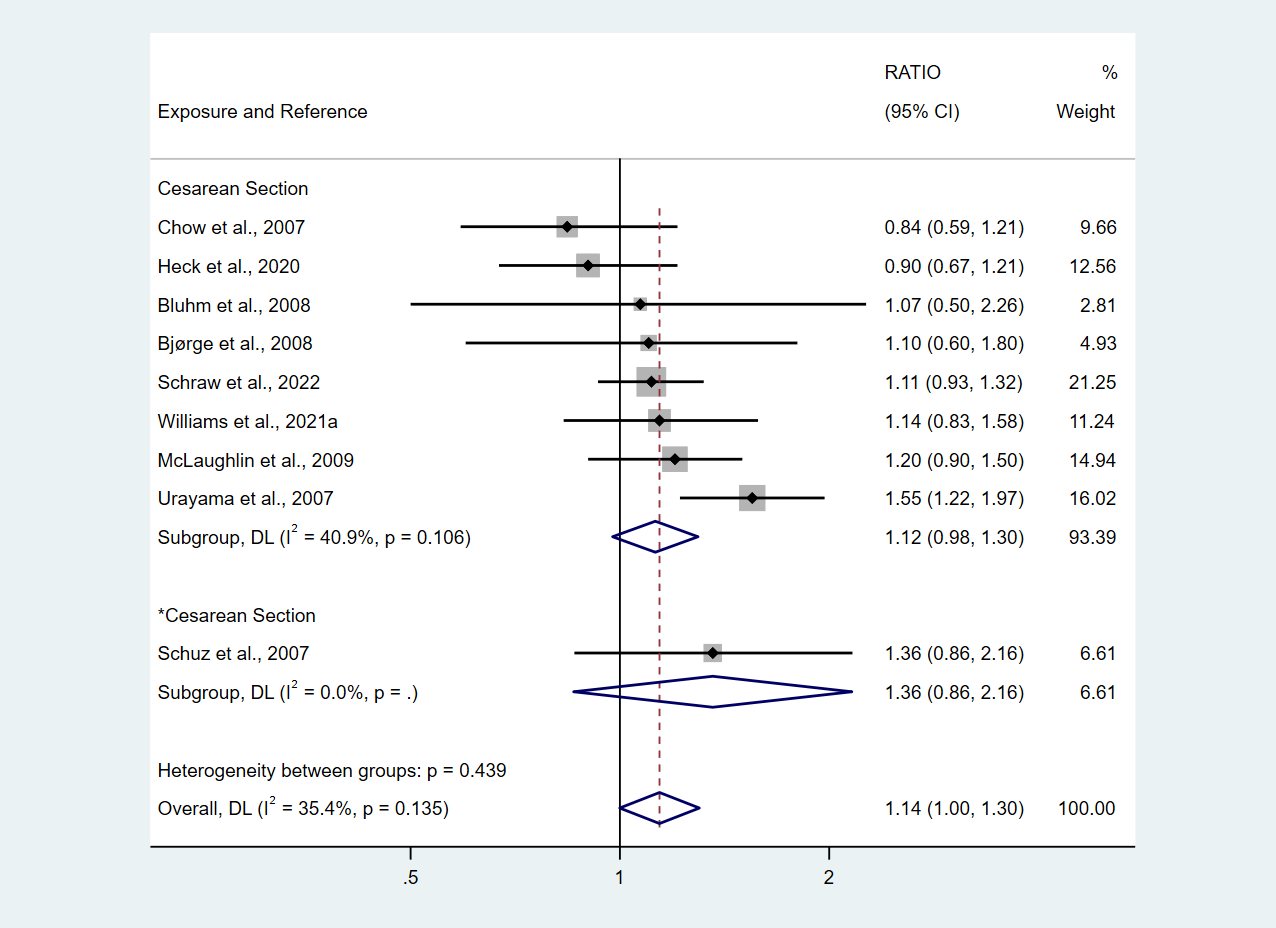


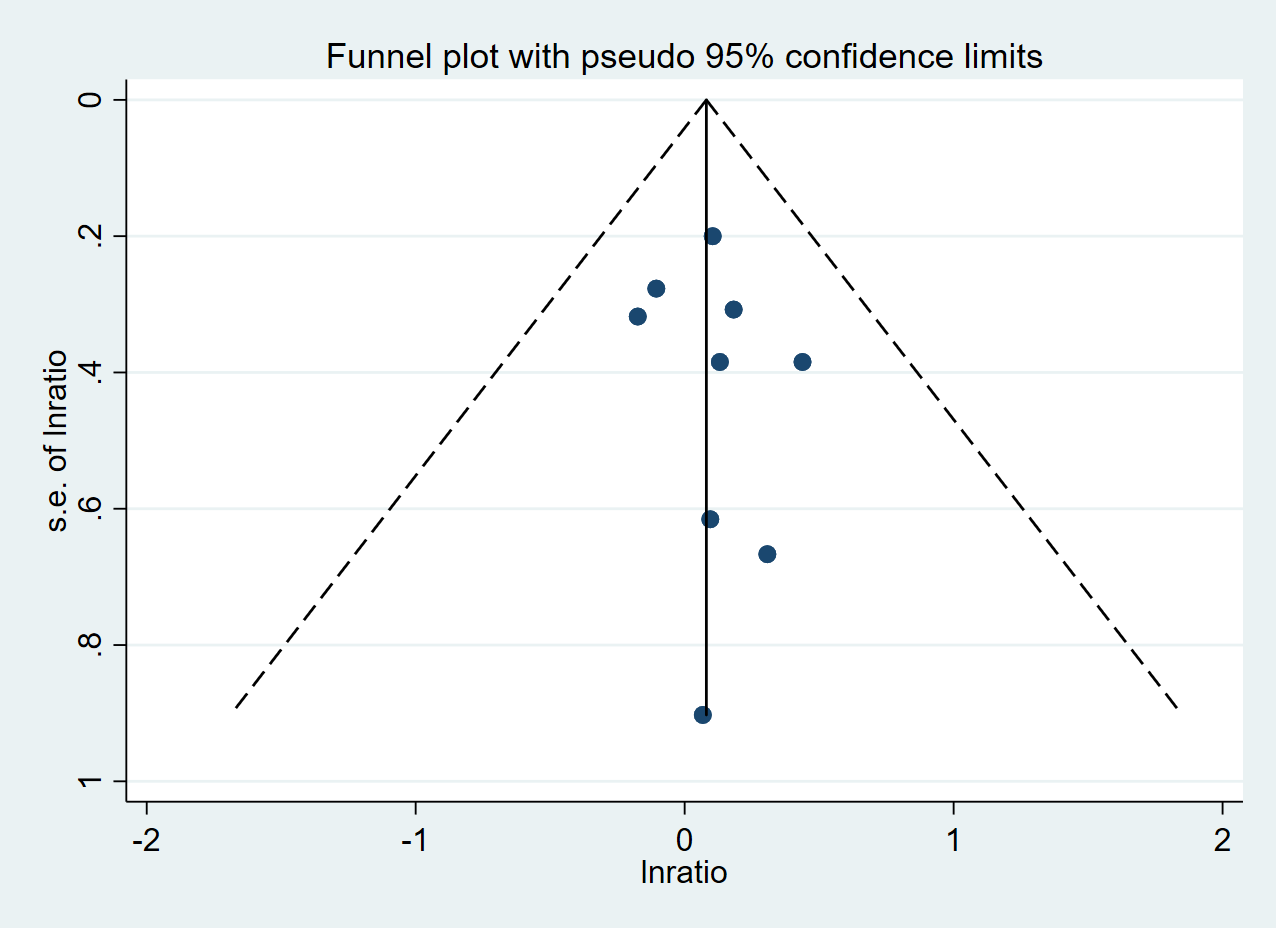


Figure S5: Forest and funnel plots of Caesarean section


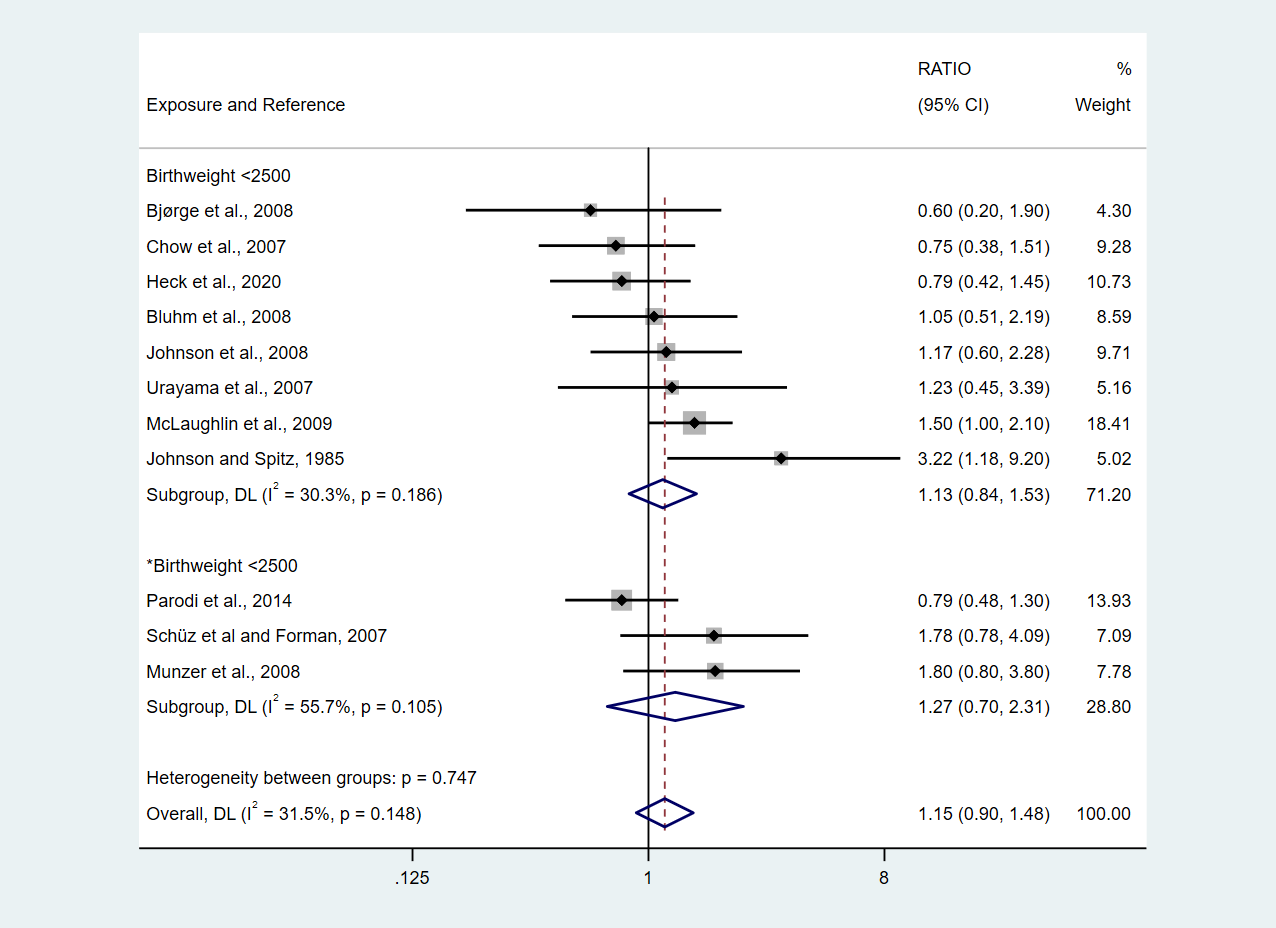


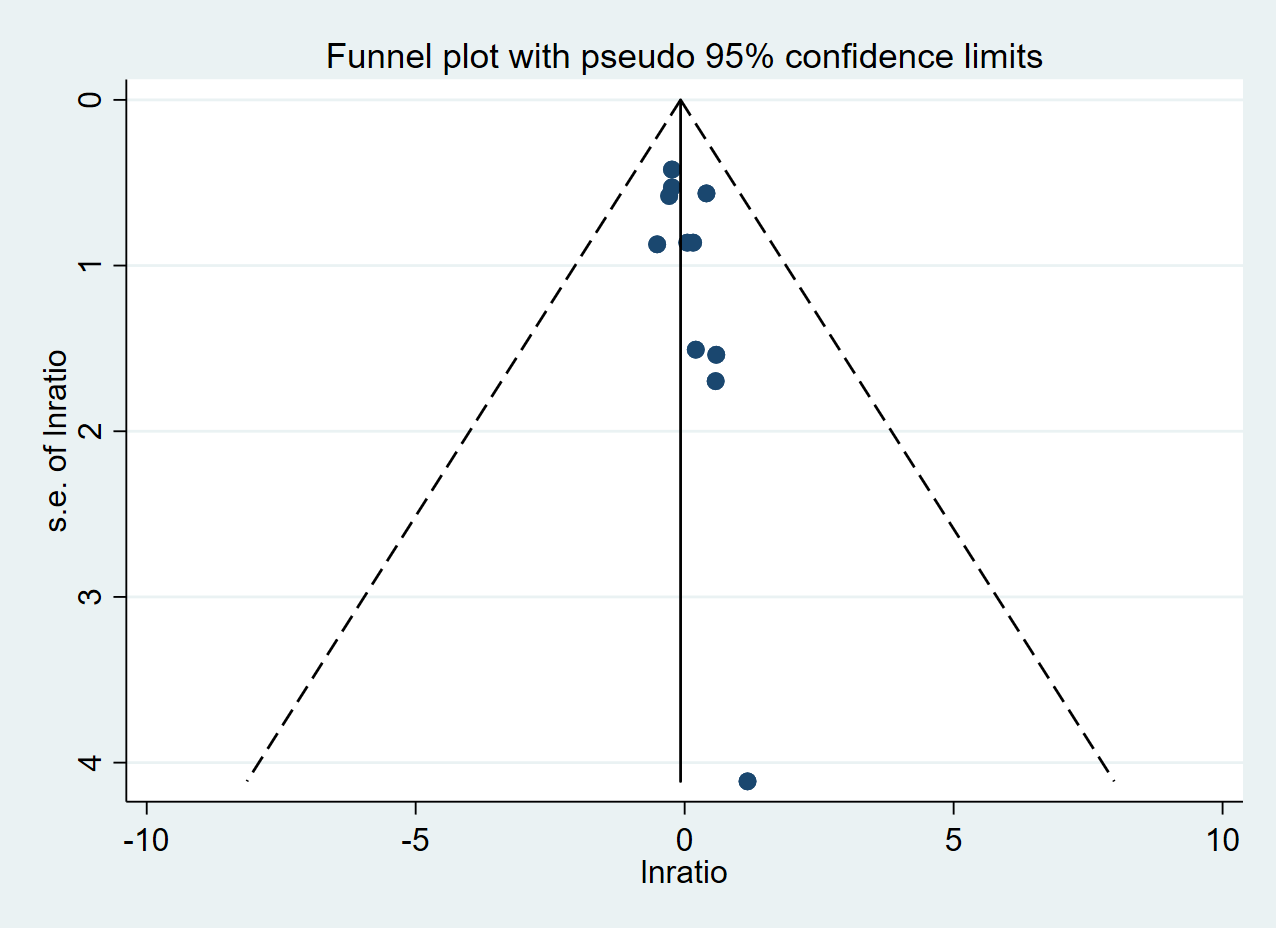


Figure S6: Forest and funnel plots of birthweight <2500 g


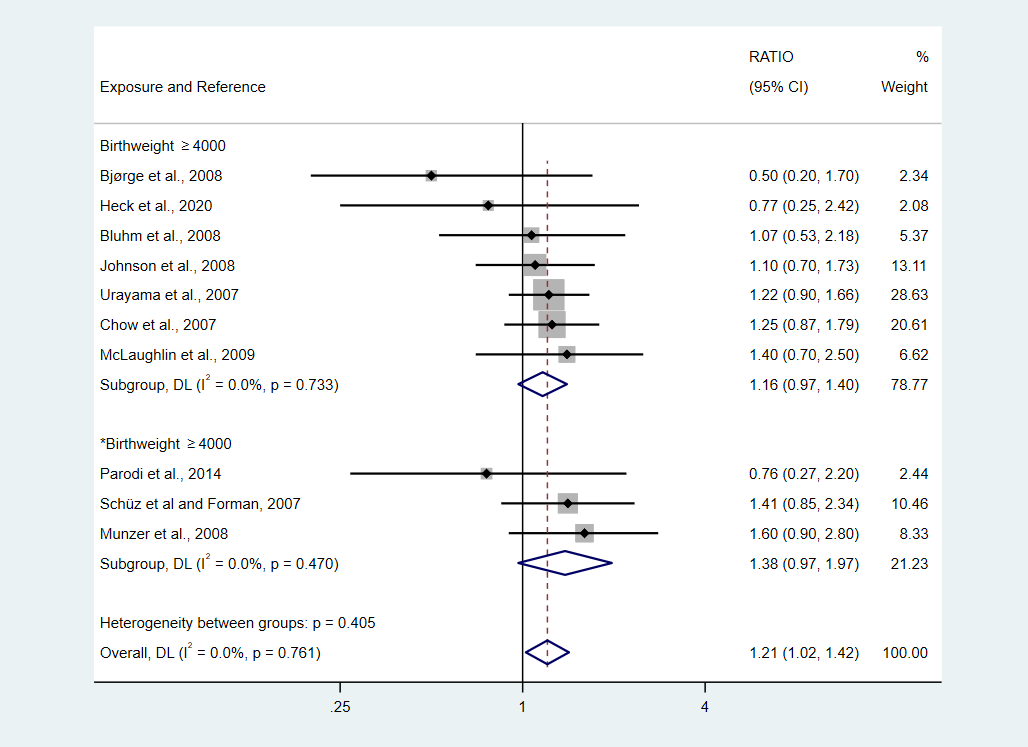


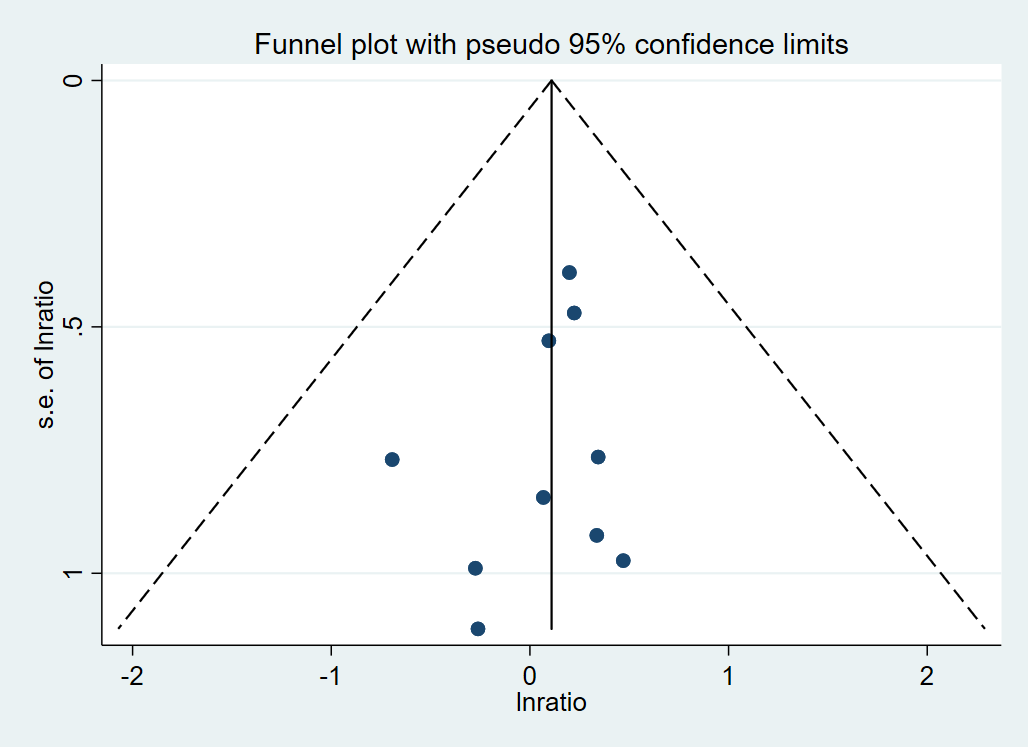


Figure S7: Forest and funnel plots of birthweight >4000 g


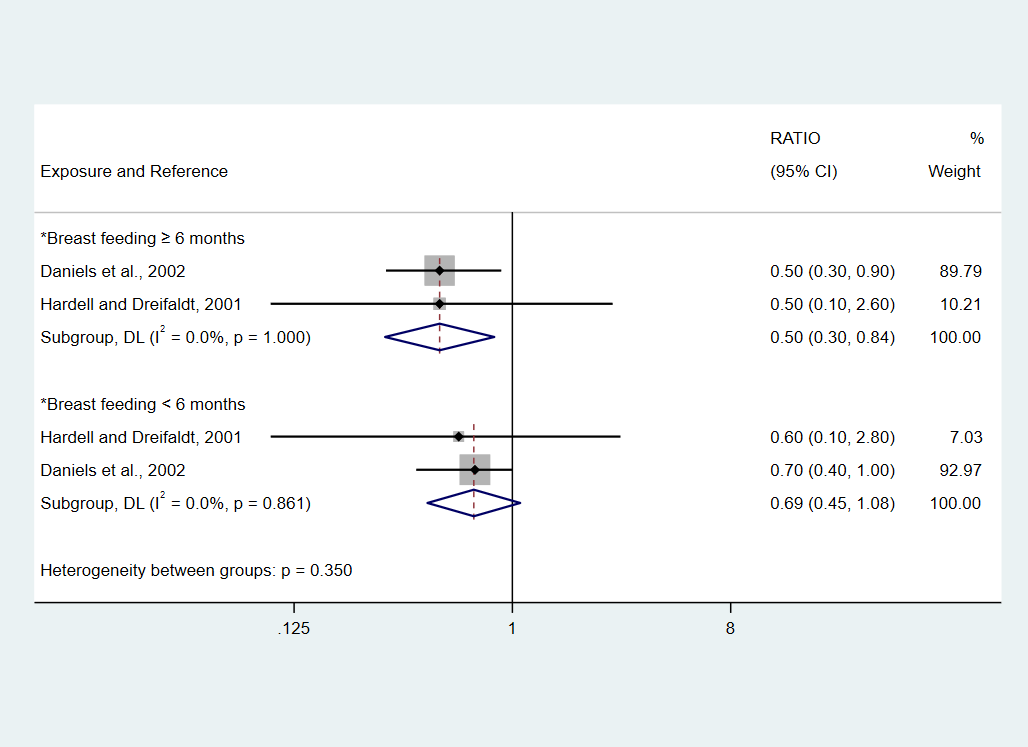


Figure S8: Forest plot of breastfeeding < and ≥ 6months


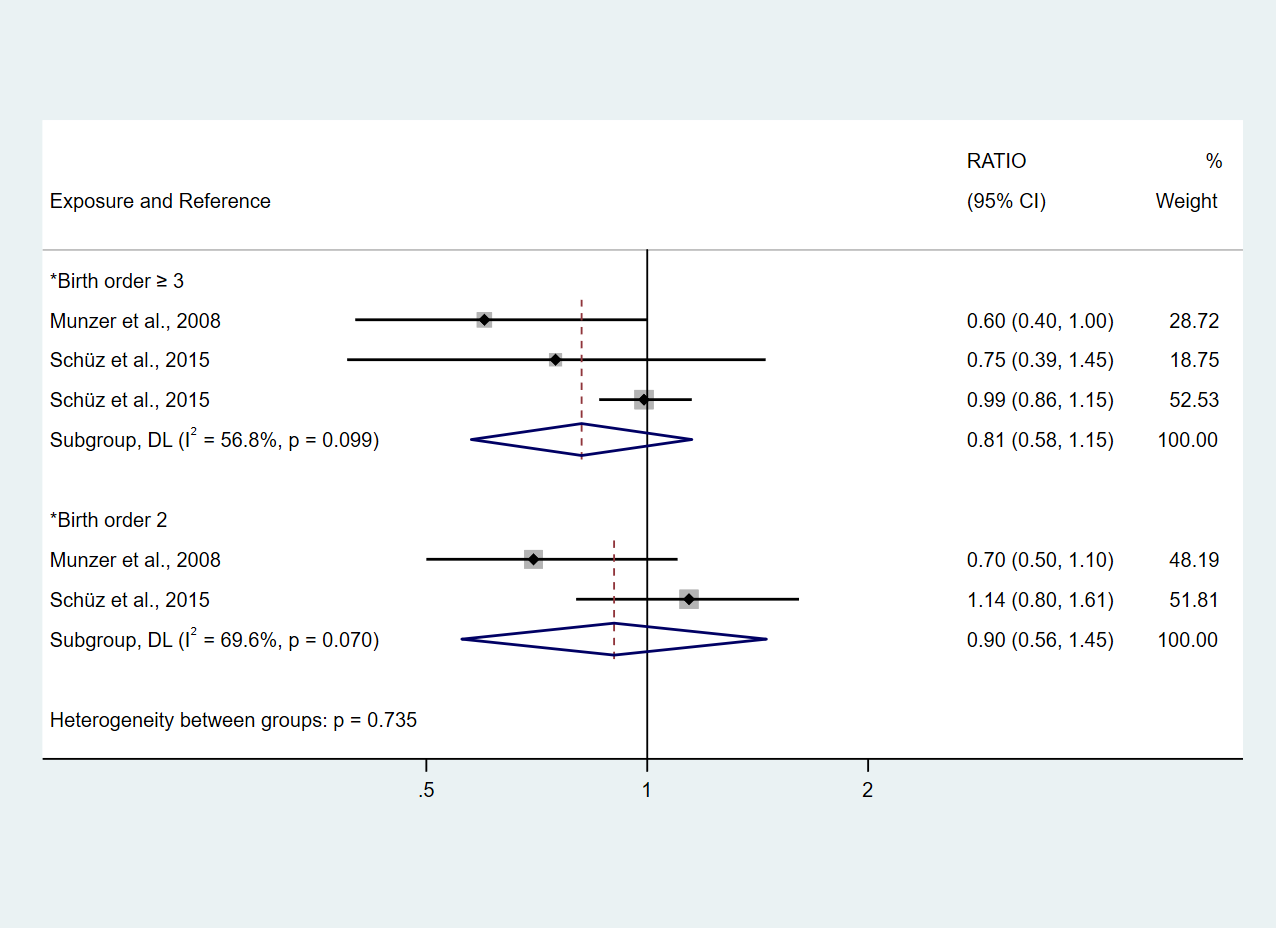


Figure S9: Forest plot of birth order 2 and 3


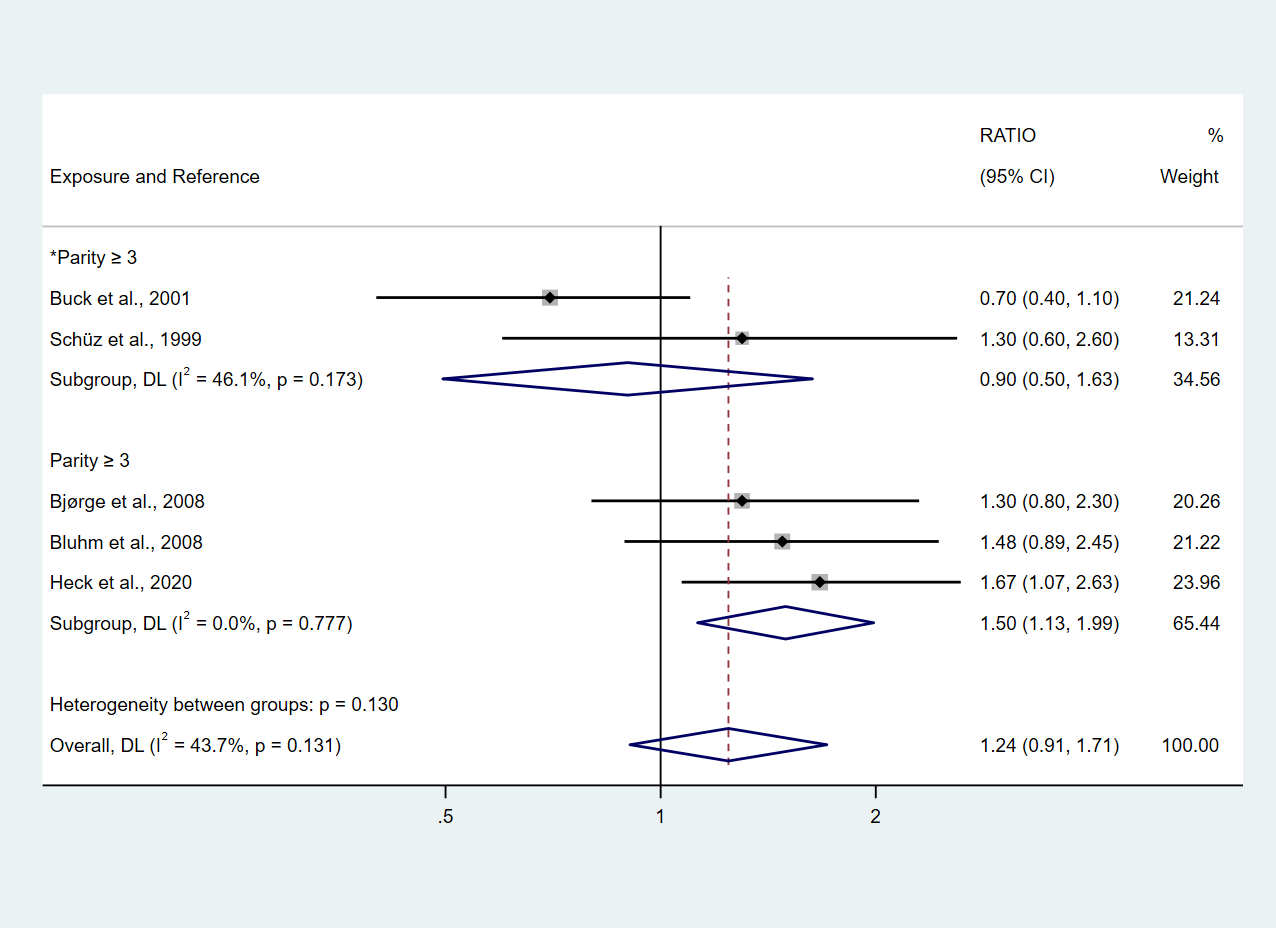


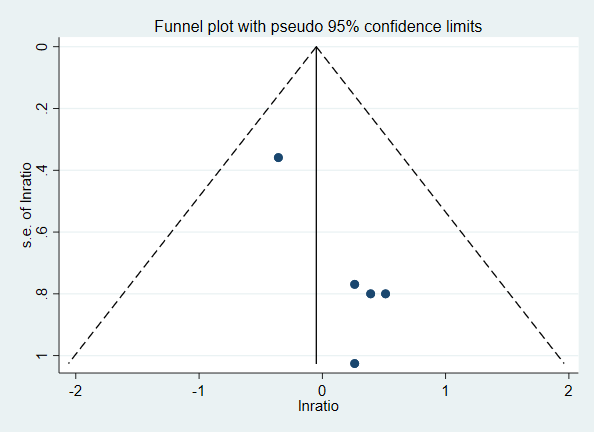


Figure S10: Forest and funnel plots of Parity ≥ 3


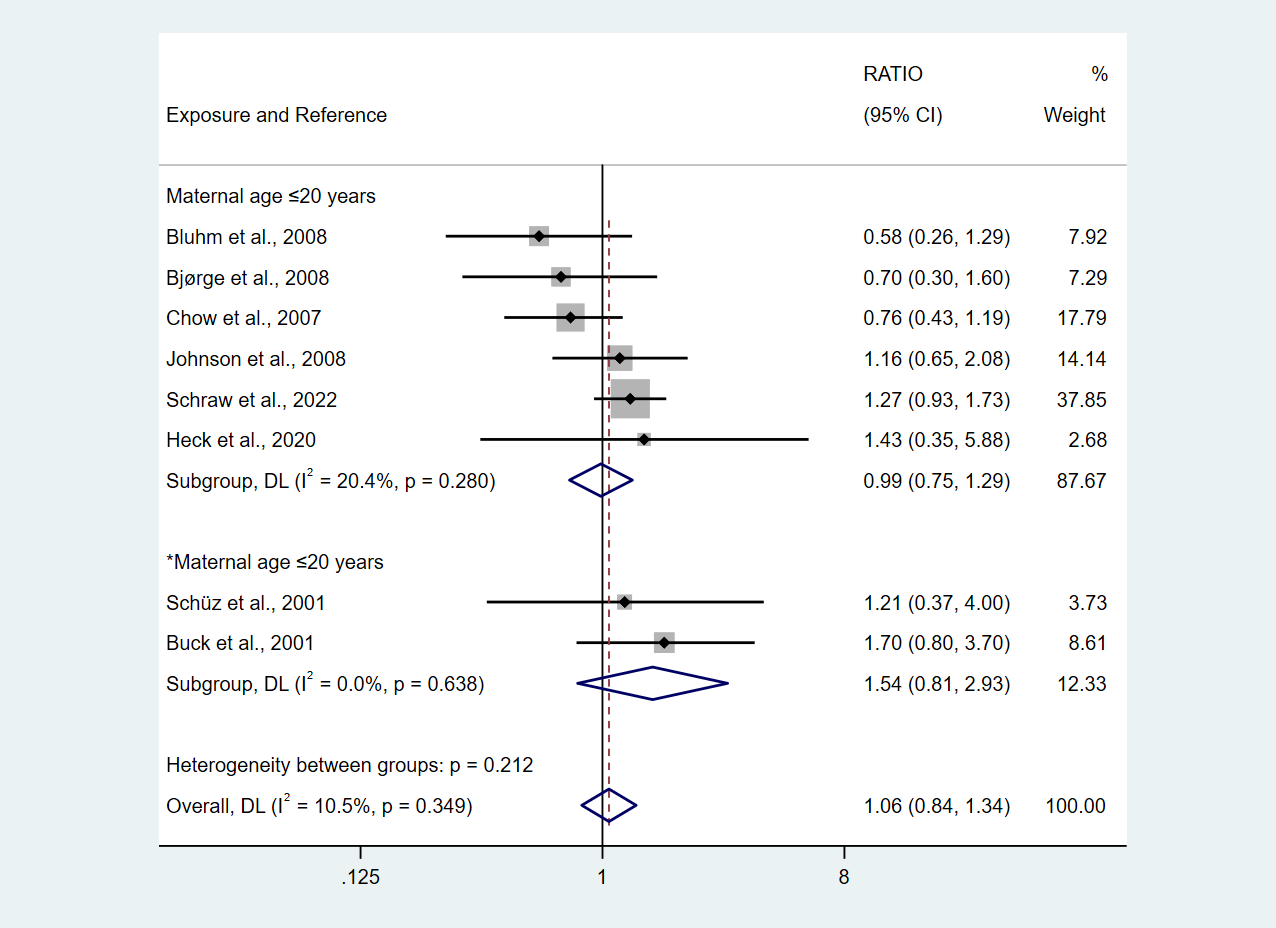


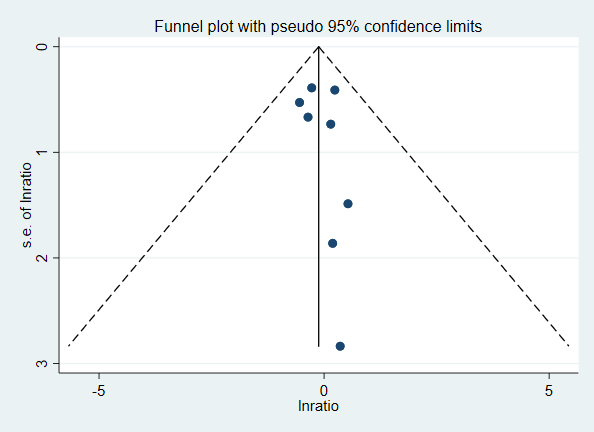


Figure S11: Forest and funnel plots of younger mothers (age <20 years)


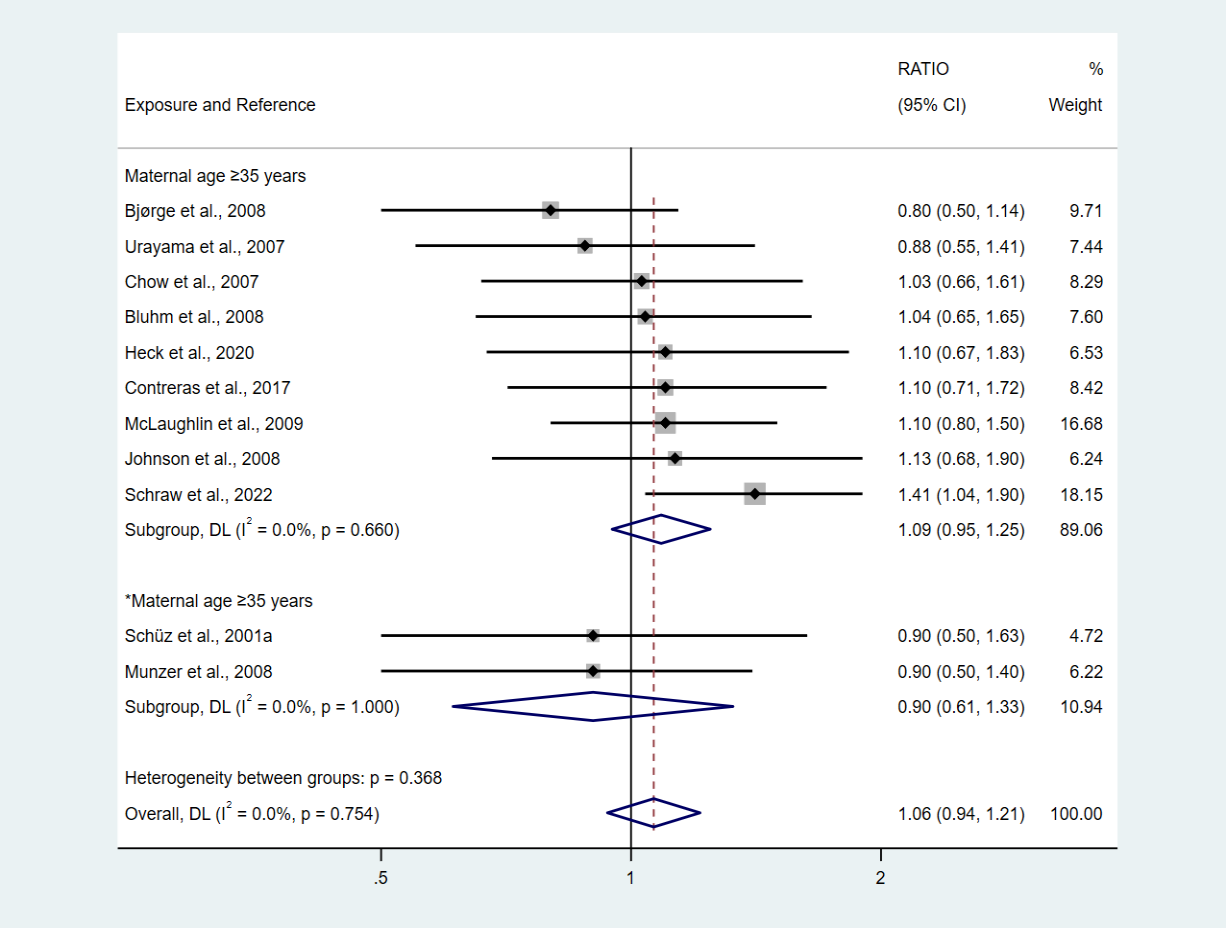


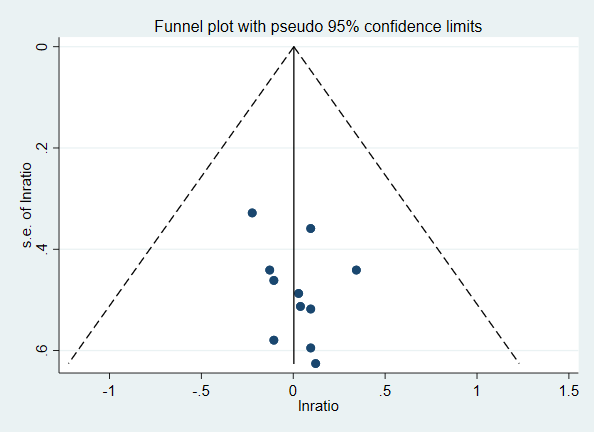


Figure S12: Forest and funnel plots of older mothers (age ≥ 35 years)


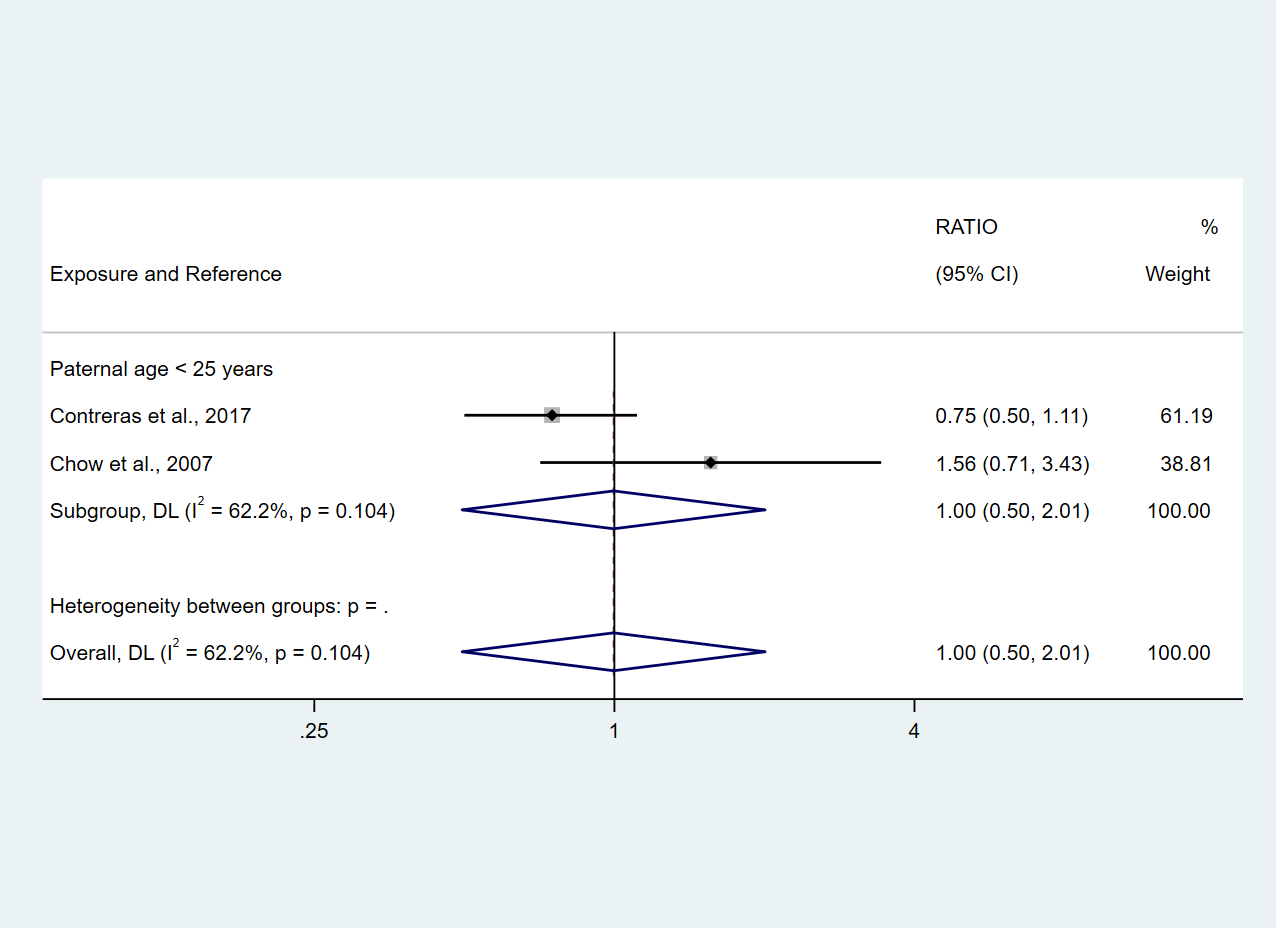


Figure S13: Forest plot of younger fathers (age <25years)


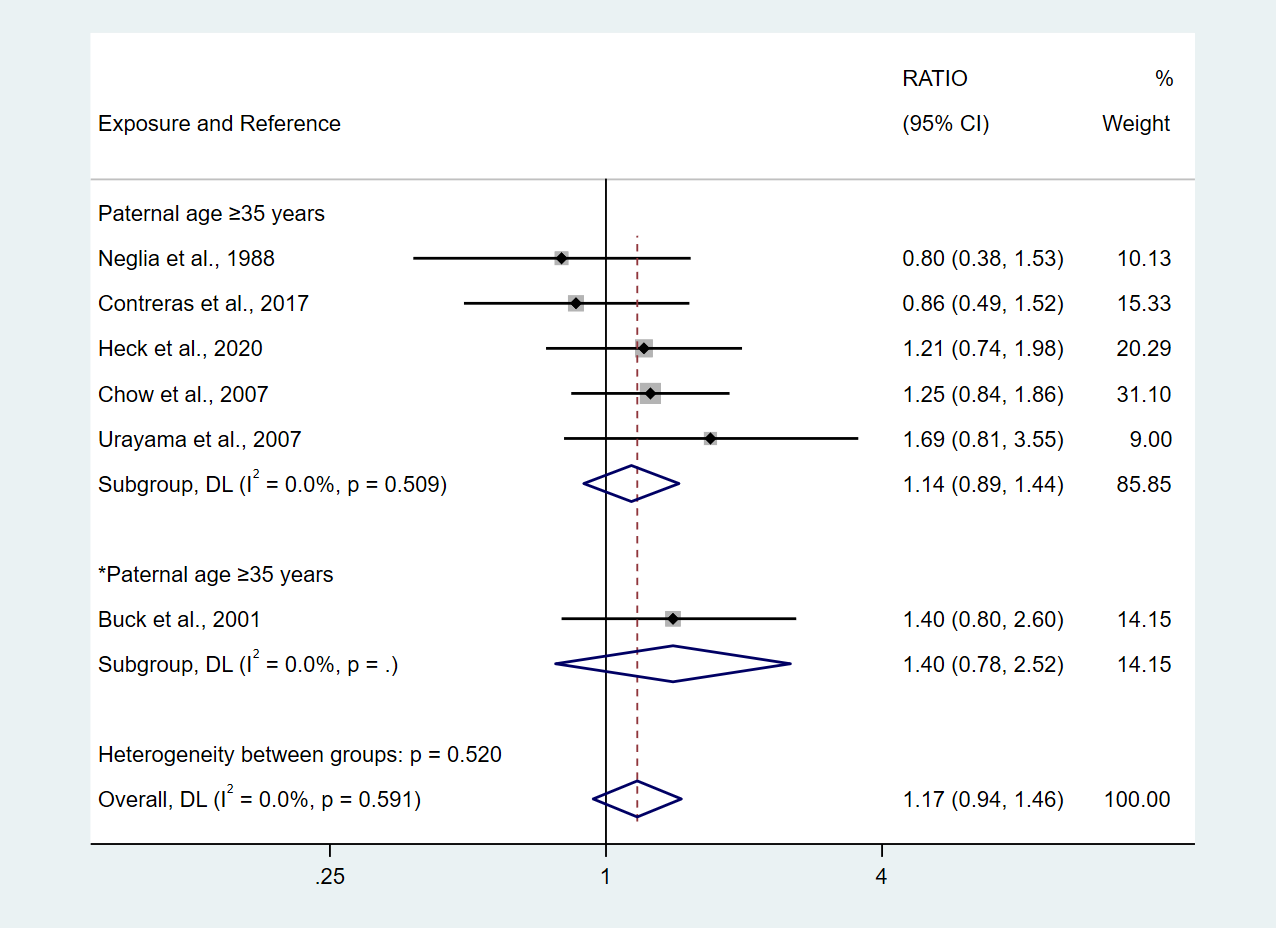


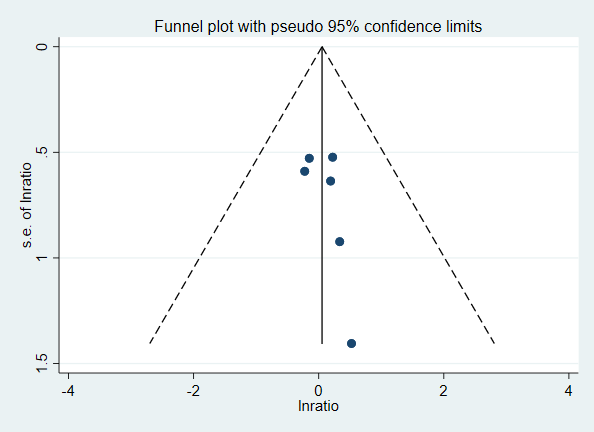


Figure S14: Forest and funnel plots of older father (age ≥ 35 years)


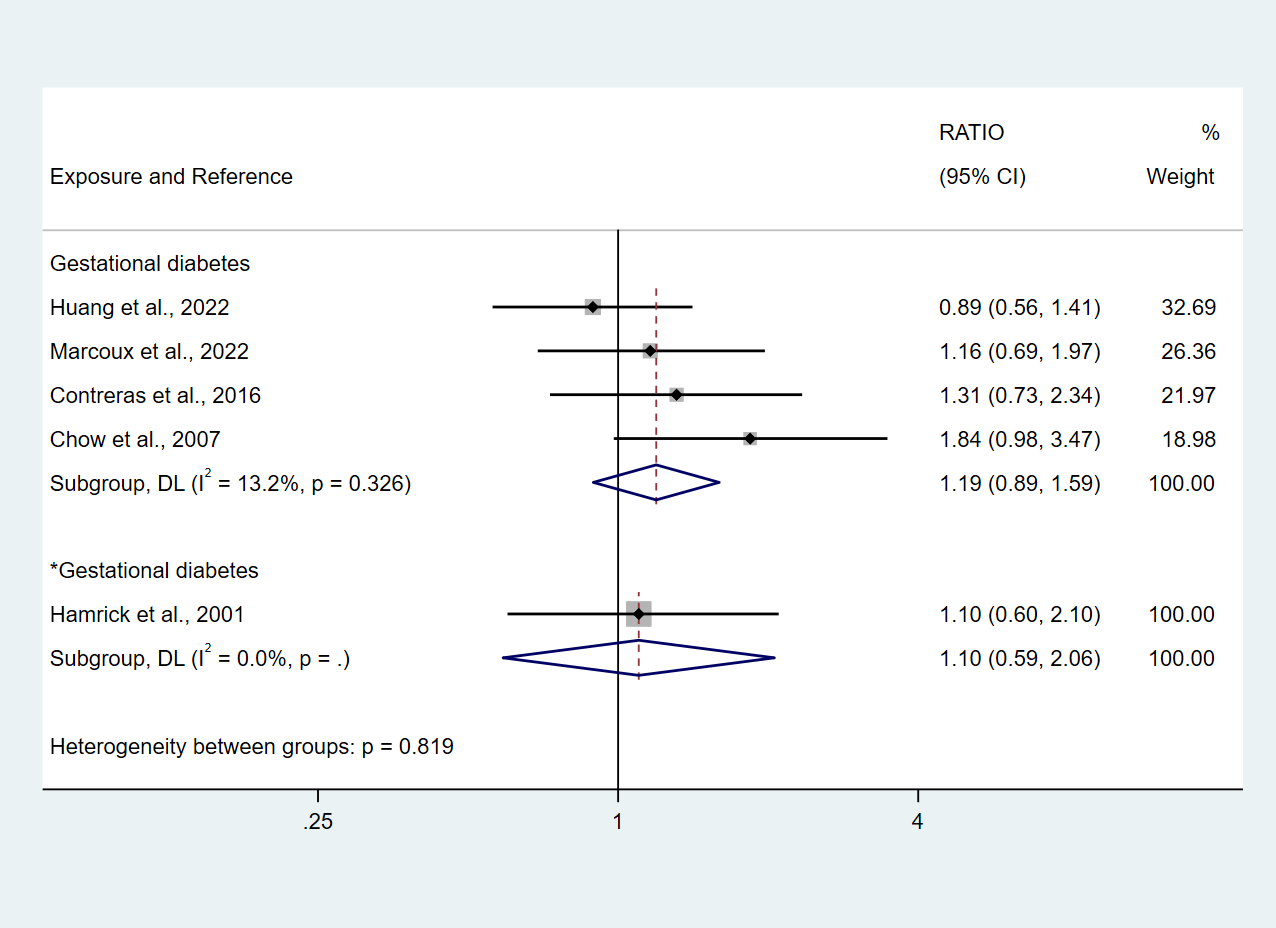


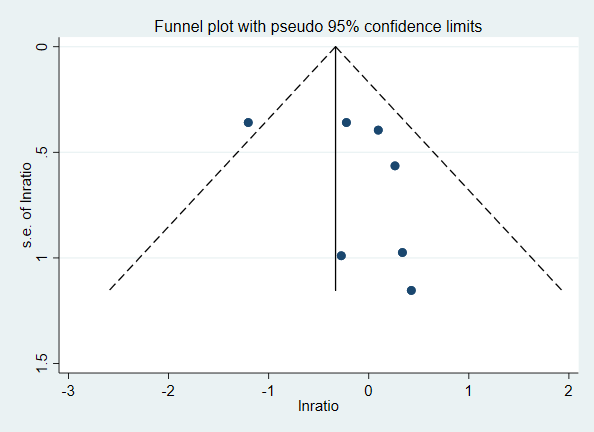


Figure S15: Forest and funnel plots of gestational diabetes


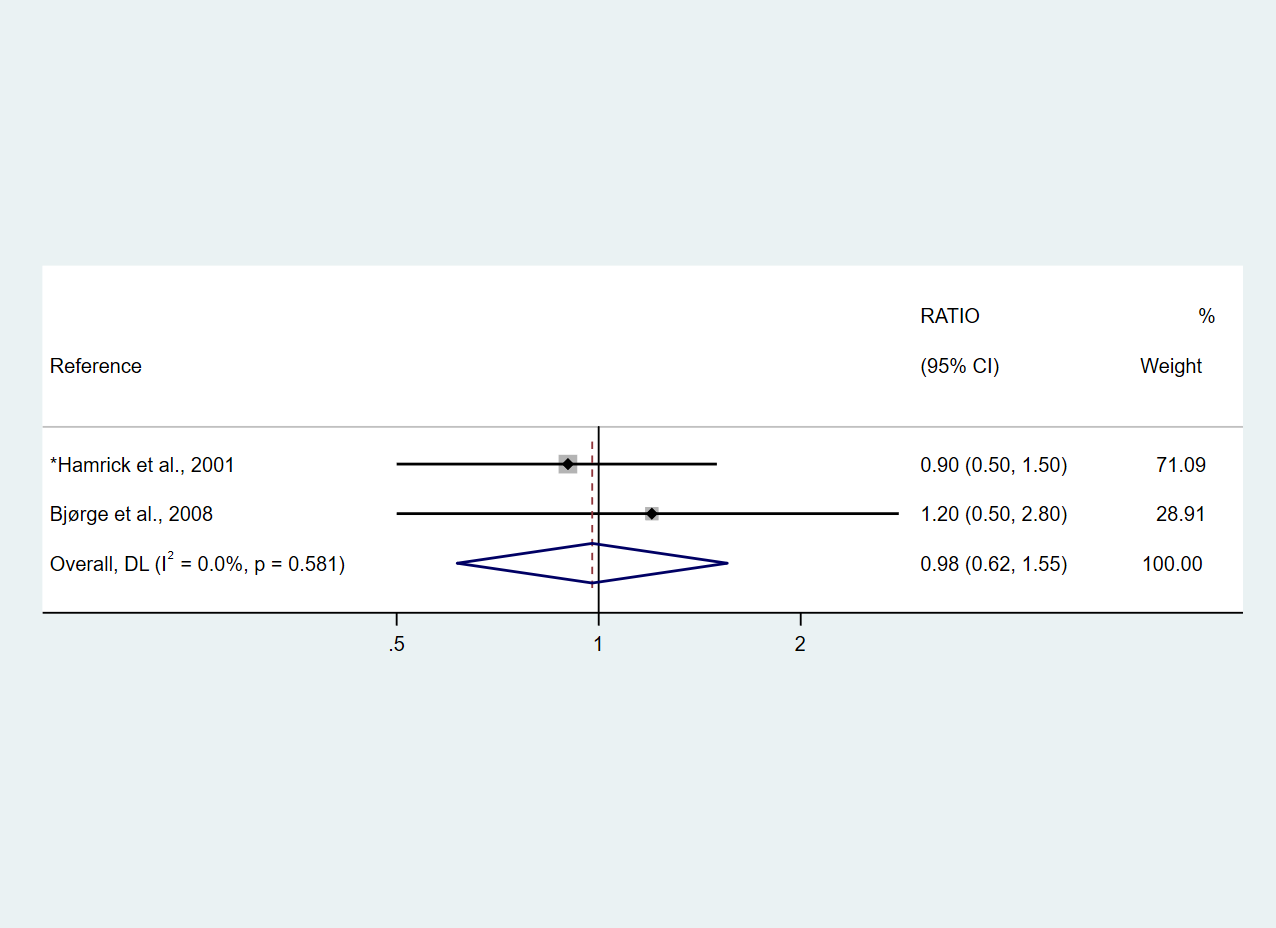


Figure S16: Forest plot of pre-eclampsia

S17- 20: Forest and funnel plots of parental lifestyle and the risk of NB


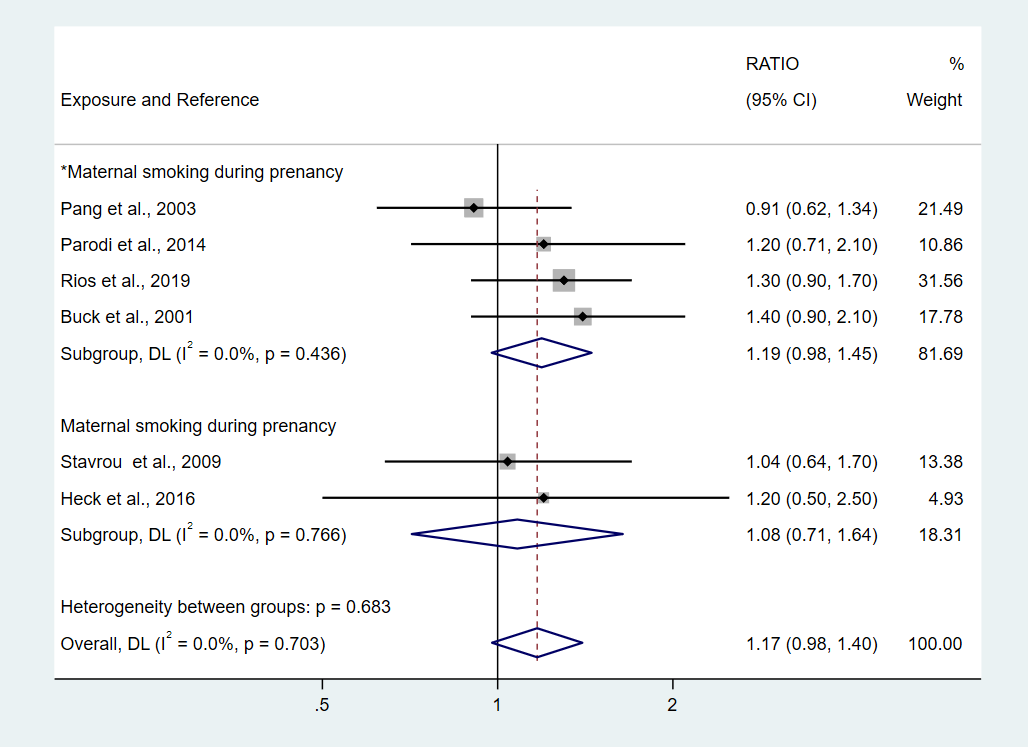


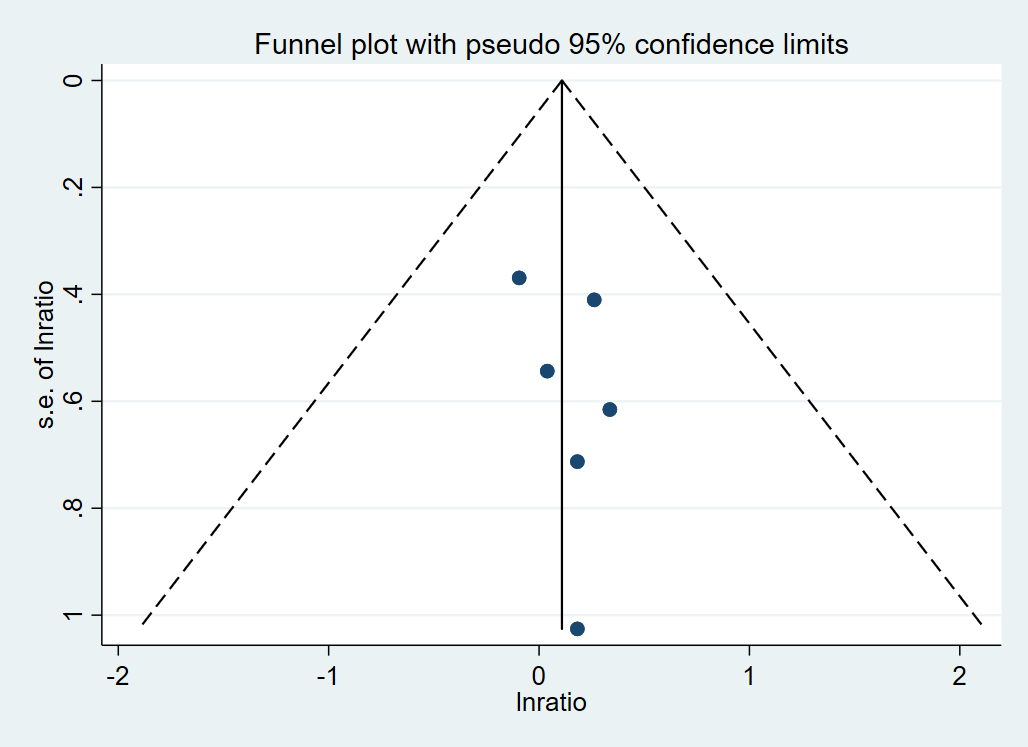


Figure S17: Forest and funnel plots of maternal smoking (ever smoking)


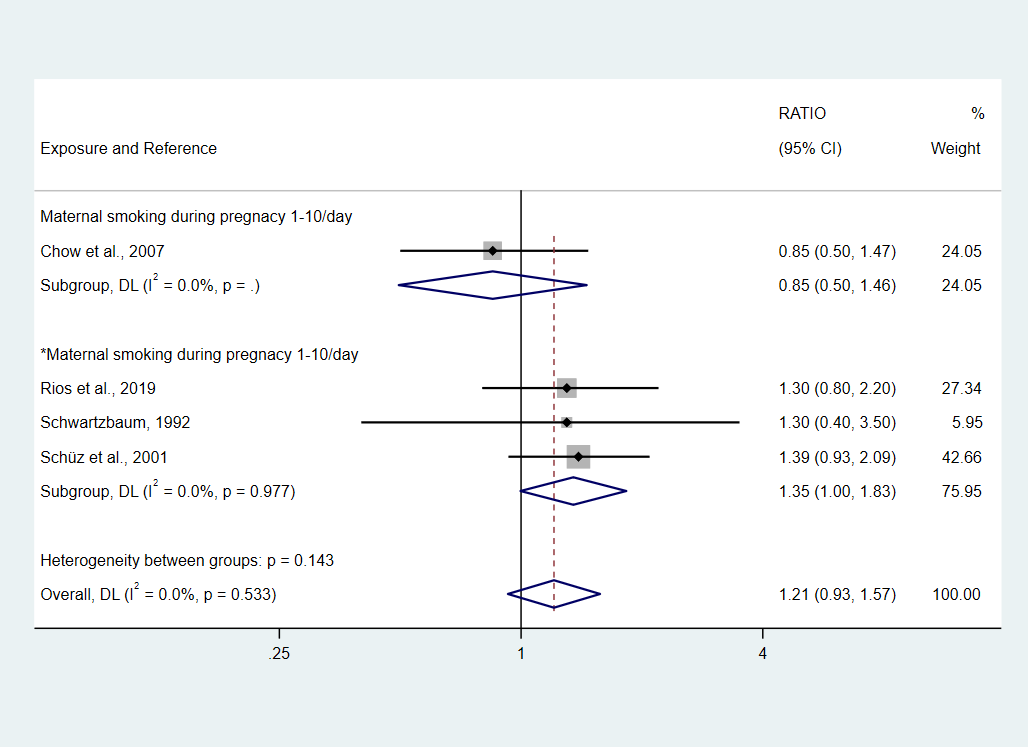


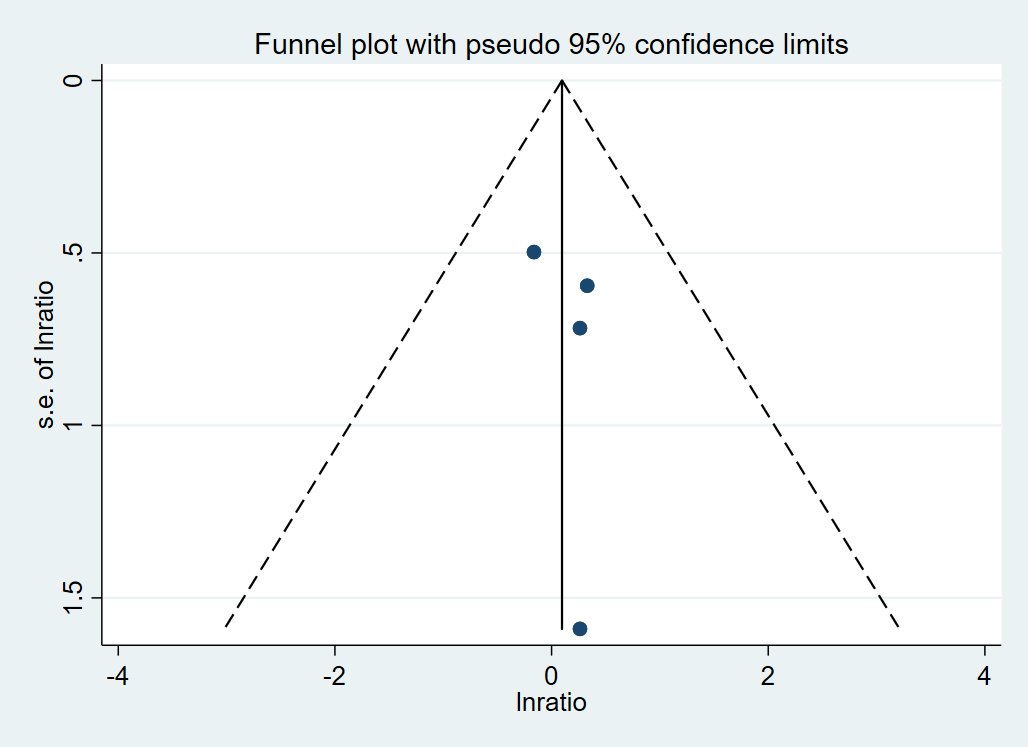


Figure S18: Forest and funnel plots of maternal smoking 1-10 cigarettes per day


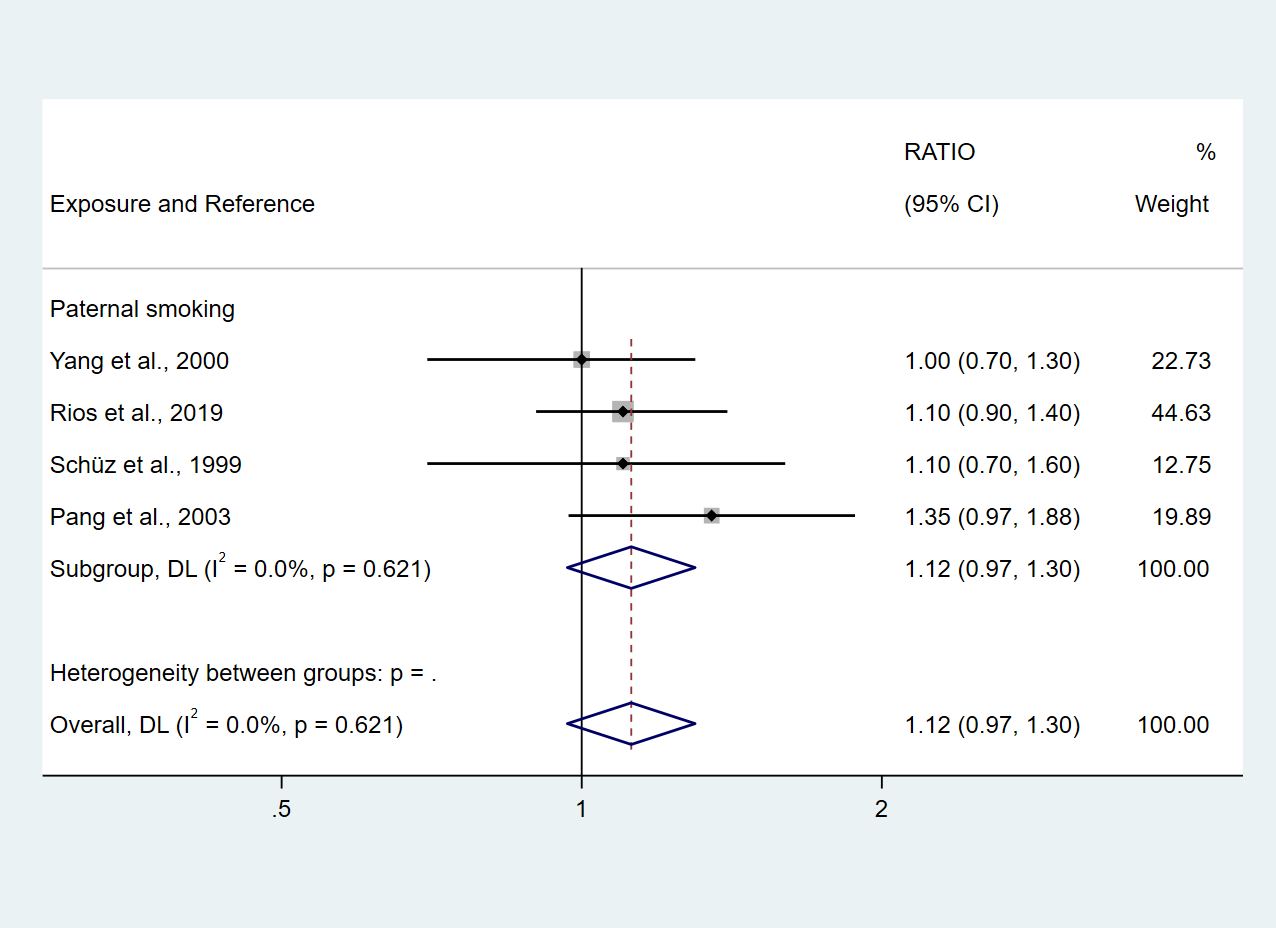


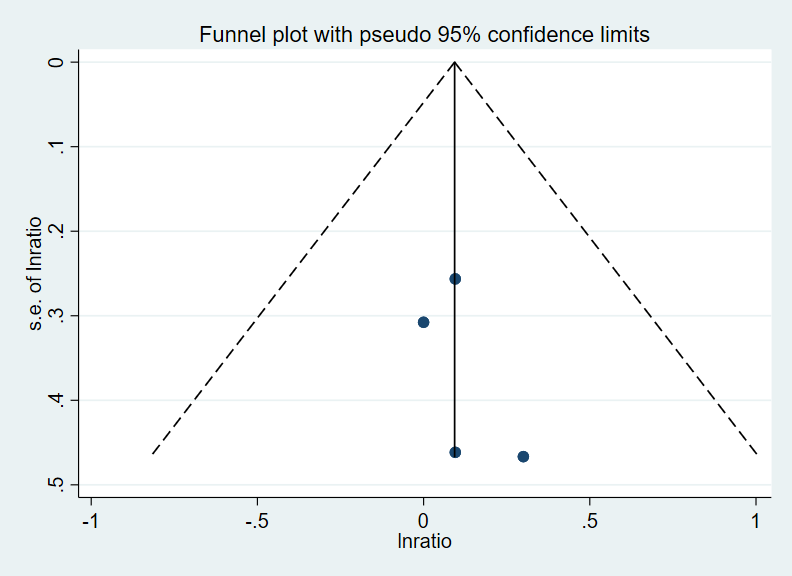


Figure S19: Forest and funnel plots of paternal smoking (ever smoking)


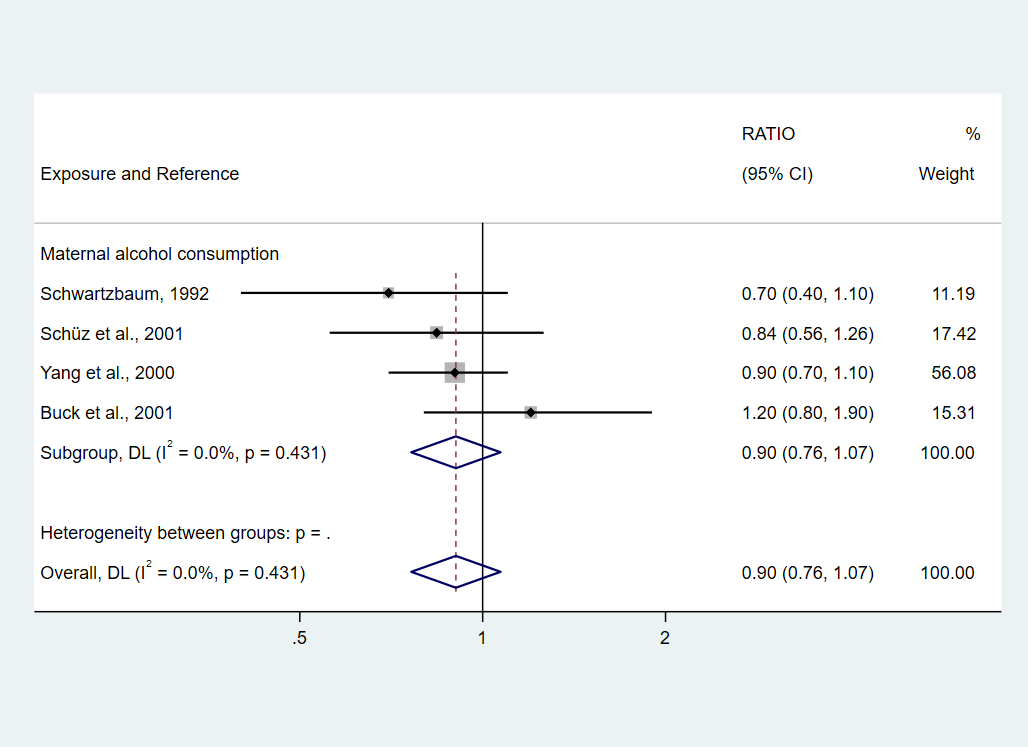


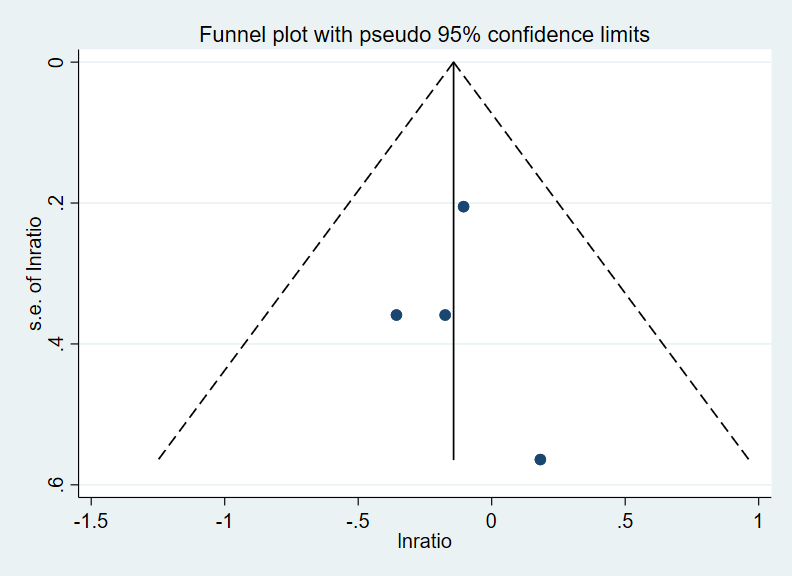


Figure S20: Forest and funnel plots of maternal alcohol consumption


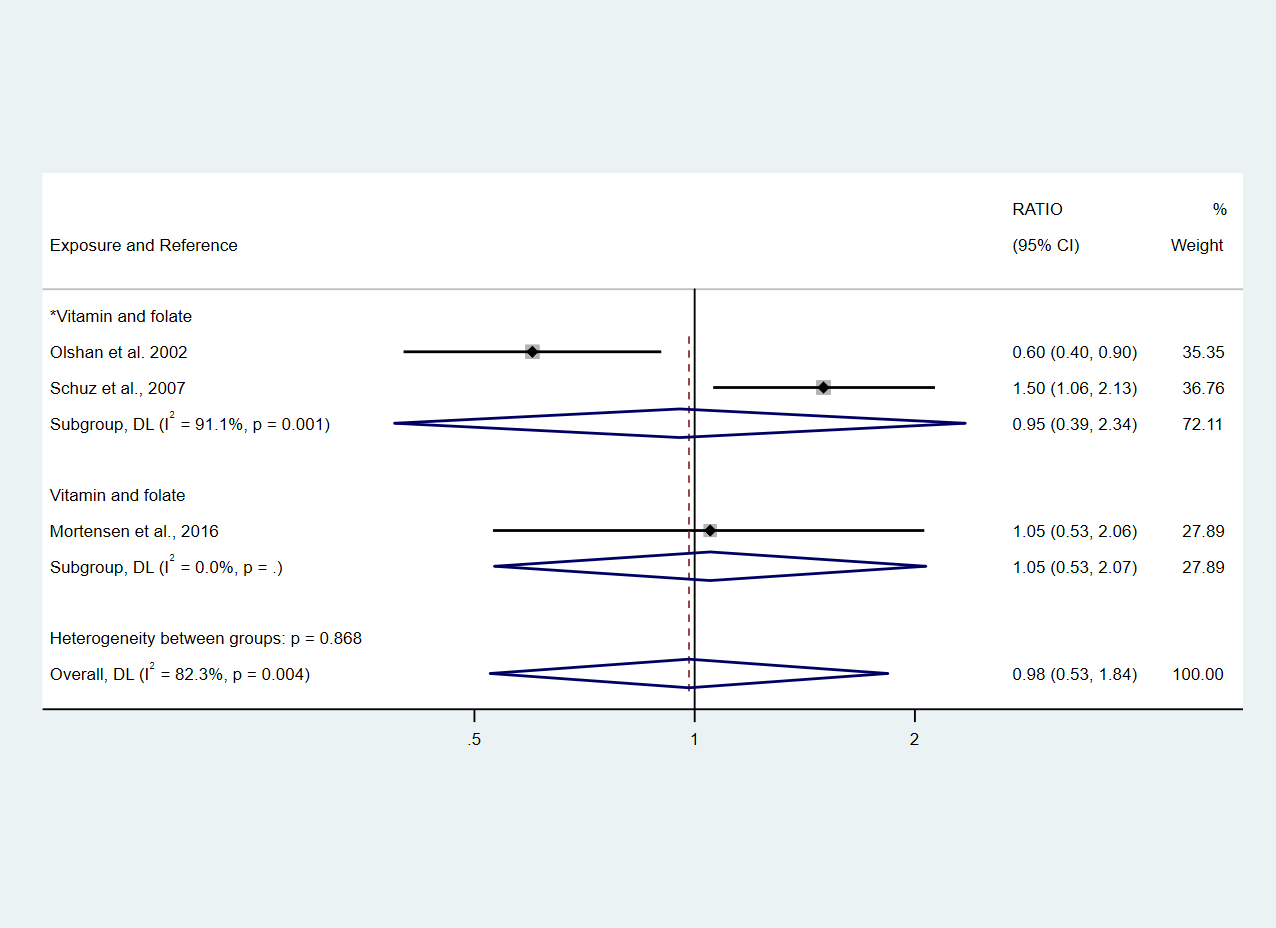


Figure S20: Forest and funnel plots of maternal vitamin and folic acid intake

**Figure S21: Forest and funnel plots of pesticides and the risk of NB**


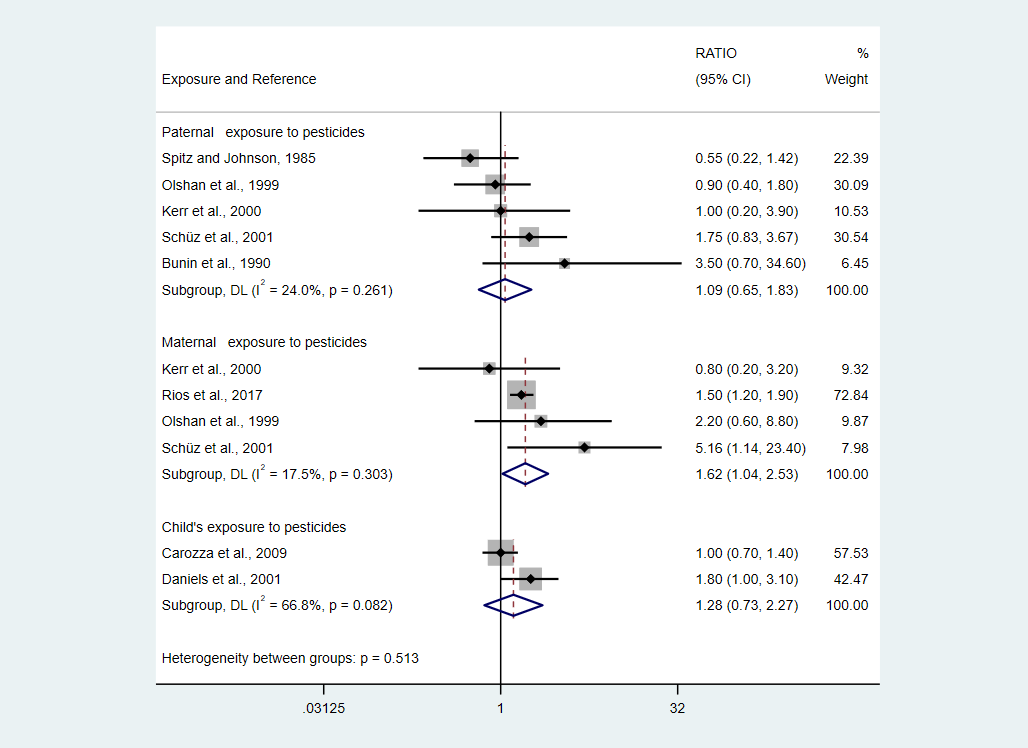


**Figure S22-24 Forest and funnel plots of parental radiation and the risk of NB**


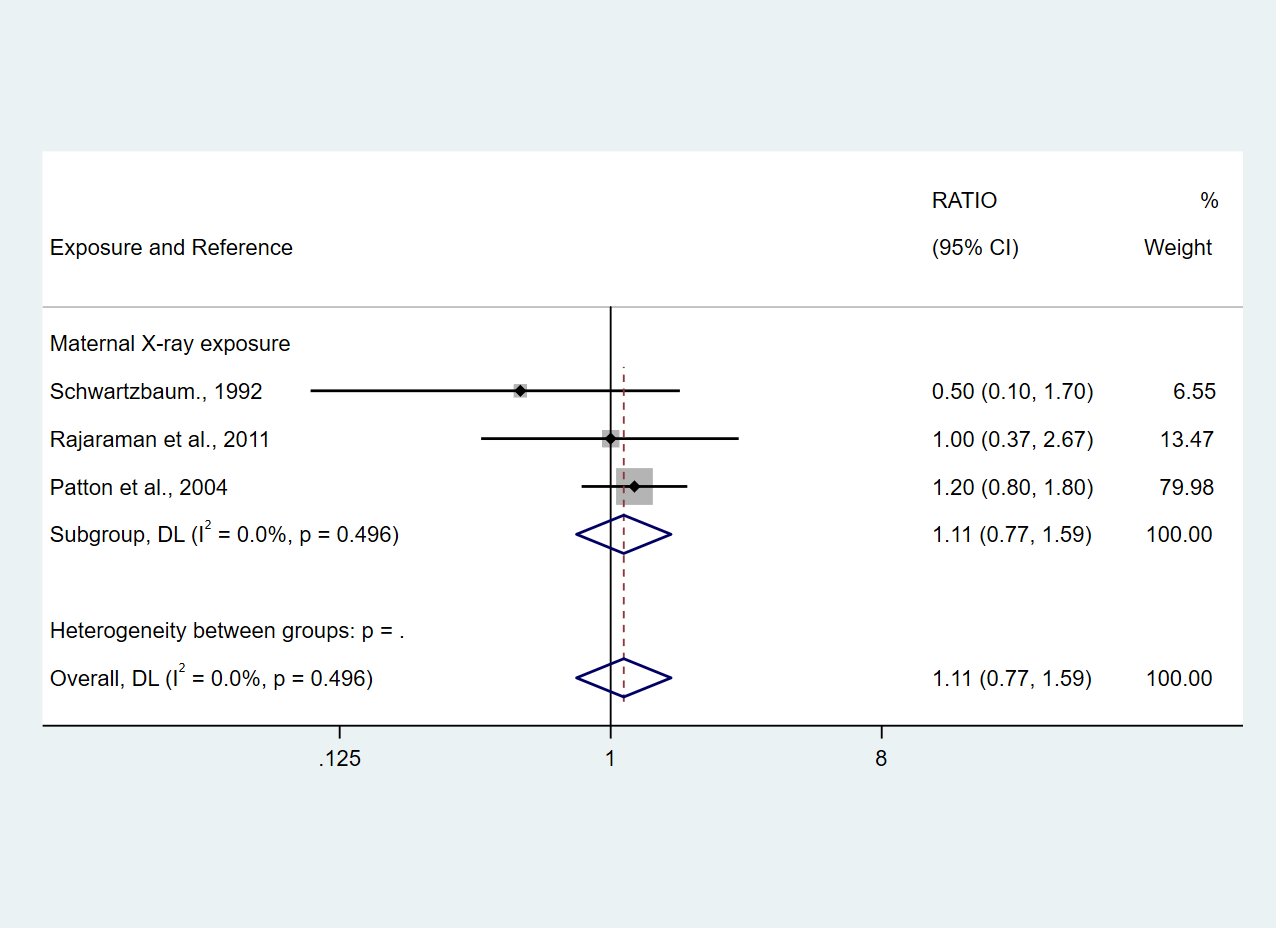


Figure S22: Forest and funnel plots of maternal exposure to X-rays and the risk of NB


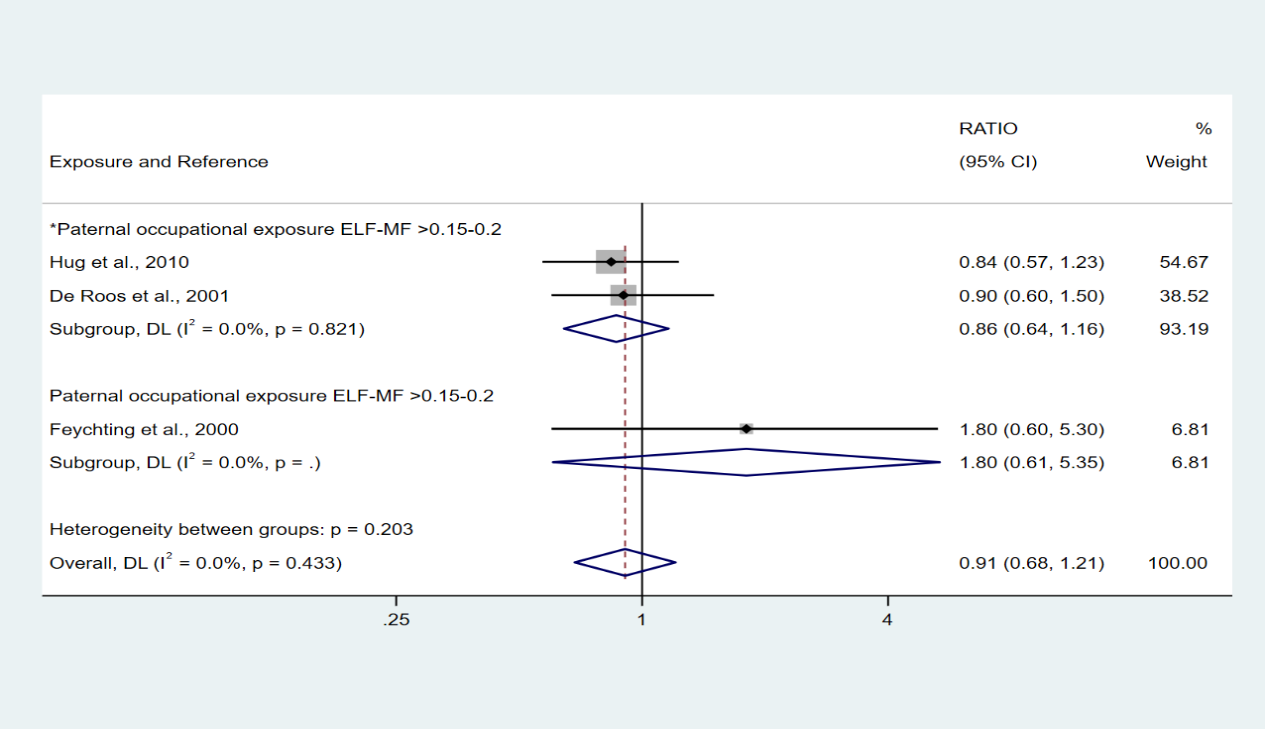


Figure S23: Forest plot of paternal exposure to ELF-MF >0.15-0.2μT and the risk of NB


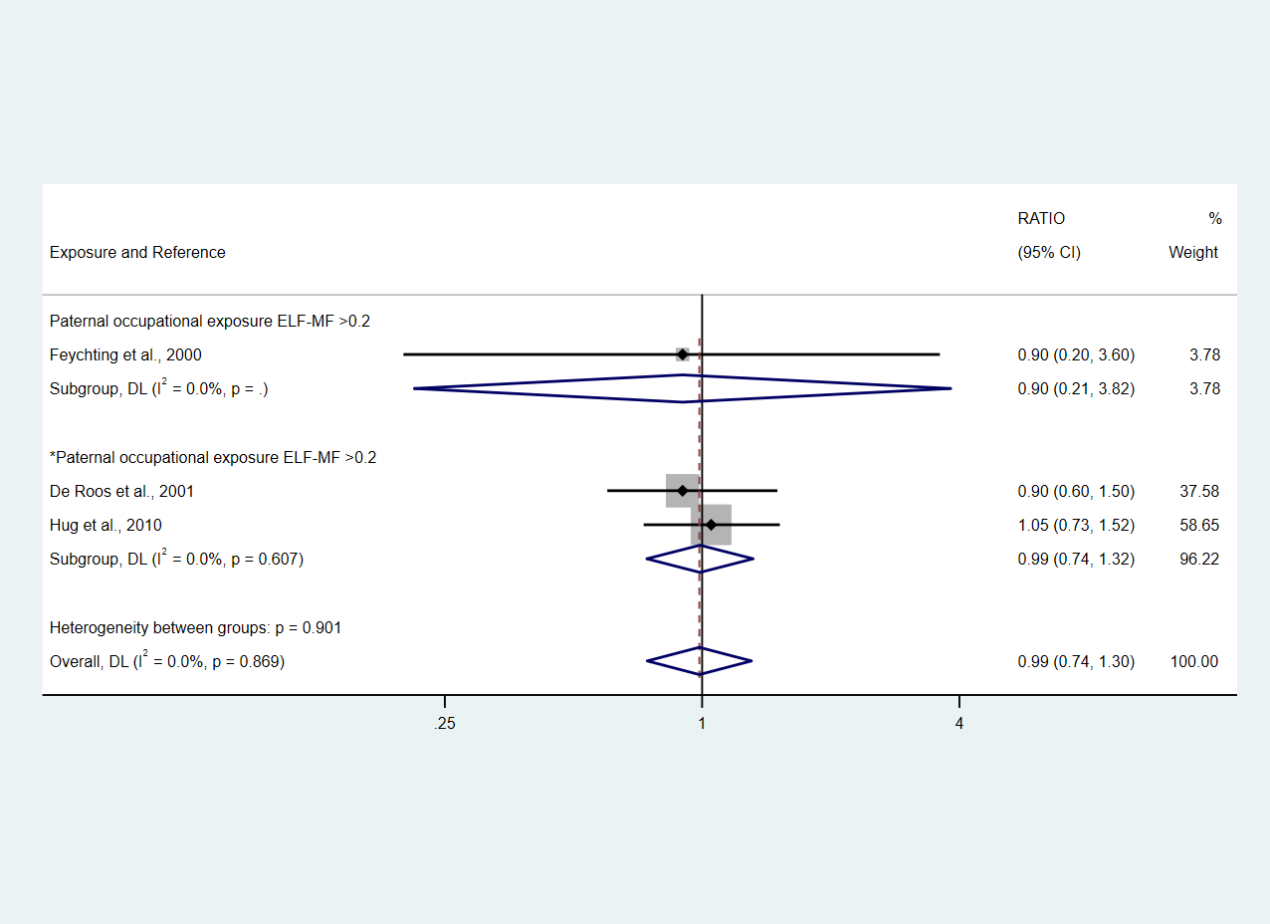


Figure S24: Forest and funnel plots of paternal exposure to ELF-MF >0.2μT and the risk of NB

**References**

1. Urayama KY, Von Behren J, Reynolds P. Birth characteristics and risk of neuroblastoma in young children. *Am J Epidemiol*. Mar 1 2007;165(5):486-95. doi:10.1093/aje/kwk041

2. Hamrick SE, Olshan AF, Neglia JP, Pollock BH. Association of pregnancy history and birth characteristics with neuroblastoma: a report from the Children's Cancer Group and the Pediatric Oncology Group. *Paediatr Perinat Epidemiol*. Oct 2001;15(4):328-37. doi:10.1046/j.1365-3016.2001.0376a.x

3. Tas ML, Reedijk AMJ, Karim-Kos HE, et al. Neuroblastoma between 1990 and 2014 in the Netherlands: Increased incidence and improved survival of high-risk neuroblastoma. *European Journal of Cancer*. 2020/01/01/ 2020;124:47-55. doi:<https://doi.org/10.1016/j.ejca.2019.09.025>

4. Irwin MS, Naranjo A, Zhang FF, et al. Revised Neuroblastoma Risk Classification System: A Report From the Children's Oncology Group. *J Clin Oncol*. Oct 10 2021;39(29):3229-3241. doi:10.1200/jco.21.00278

5. Brodeur GM. Spontaneous regression of neuroblastoma. *Cell Tissue Res*. May 2018;372(2):277-286. doi:10.1007/s00441-017-2761-2

6. Sorahan T, Lancashire RJ, Hulten MA, Peck I, Stewart AM. Childhood cancer and parental use of tobacco: Deaths from 1953 to 1955. *British Journal of Cancer*. 1997;75(1):134-138. doi:10.1038/bjc.1997.22

7. Tsai J, Kaye WE, Bove FJ. Wilms’ tumor and exposures to residential and occupational hazardous chemicals. *International Journal of Hygiene and Environmental Health*. 2006/01/10/ 2006;209(1):57-64. doi:<https://doi.org/10.1016/j.ijheh.2005.09.003>

8. Yeazel MW, Ross JA, Buckley JD, Woods WG, Ruccione K, Robison LL. High birth weight and risk of specific childhood cancers: A report from the Children's Cancer Group. *Journal of Pediatrics*. Nov 1997;131(5):671-677. doi:10.1016/s0022-3476(97)70091-x

9. Daniels JL, Olshan AF, Teschke K, et al. Residential pesticide exposure and neuroblastoma. *Epidemiology*. Jan 2001;12(1):20-27. doi:10.1097/00001648-200101000-00005

10. Bjørge T, Engeland A, Tretli S, Heuch I. Birth and parental characteristics and risk of neuroblastoma in a population-based Norwegian cohort study. *Br J Cancer*. Oct 7 2008;99(7):1165-9. doi:10.1038/sj.bjc.6604646

11. Chow EJ, Friedman DL, Mueller BA. Maternal and perinatal characteristics in relation to neuroblastoma. *Cancer*. Mar 1 2007;109(5):983-92. doi:10.1002/cncr.22486

12. Carozza SE, Li B, Wang Q, Horel S, Cooper S. Agricultural pesticides and risk of childhood cancers. *INTERNATIONAL JOURNAL OF HYGIENE AND ENVIRONMENTAL HEALTH*. MAR 2009;212(2):186-195. doi:10.1016/j.ijheh.2008.06.002

13. Schuz J, Kaletsch U, Meinert R, Kaatsch P, Spix C, Michaelis J. Risk factors for neuroblastoma at different stages of disease. Results from a population-based case-control study in Germany. *Journal of Clinical Epidemiology*. Jul 2001;54(7):702-709. doi:10.1016/s0895-4356(00)00339-5

14. Yang Q, Olshan AF, Bondy ML, et al. Parental smoking and alcohol consumption and risk of neuroblastoma. *Cancer Epidemiol Biomarkers Prev*. Sep 2000;9(9):967-72.

15. Page MJ, McKenzie JE, Bossuyt PM, et al. The PRISMA 2020 statement: an updated guideline for reporting systematic reviews. *BMJ*. 2021;372:n71. doi:10.1136/bmj.n71

16. Onyije FM, Dolatkhah R, Olsson A, et al. Risk factors for childhood brain tumours: A systematic review and meta-analysis of observational studies from 1976 to 2022. *Cancer Epidemiology*. 2024/02/01/ 2024;88:102510. doi:<https://doi.org/10.1016/j.canep.2023.102510>

17. Onyije FM, Dolatkhah R, Olsson A, Bouaoun L, Schüz J. Environmental risk factors of Wilms tumour: A systematic review and meta-analysis. *EJC Paediatric Oncology*. 2024/12/01/ 2024;4:100178. doi:<https://doi.org/10.1016/j.ejcped.2024.100178>

18. *Cochrane Handbook for Systematic Reviews of Interventions version 6.4* Higgins JPT, Thomas J, Chandler J, Cumpston M, Li T, Page MJ, Welch VA ed. vol version 6.4 (updated August 2023).

19. Moola S, Munn Z, Tufanaru C, et al. Chapter 7: Systematic reviews of etiology and risk. In: Aromataris E, Munn Z (Editors) *JBI Manual for Evidence Synthesis*. 2020;

20. Egger M, Davey Smith G, Schneider M, Minder C. Bias in meta-analysis detected by a simple, graphical test. *Bmj*. Sep 13 1997;315(7109):629-34. doi:10.1136/bmj.315.7109.629

21. Lin L, Shi L, Chu H, Murad MH. The magnitude of small-study effects in the Cochrane Database of Systematic Reviews: an empirical study of nearly 30 000 meta-analyses. *BMJ Evid Based Med*. Feb 2020;25(1):27-32. doi:10.1136/bmjebm-2019-111191

22. Munzer C, Menegaux F, Lacour B, et al. Birth-related characteristics, congenital malformation, maternal reproductive history and neuroblastoma: the ESCALE study (SFCE). *Int J Cancer*. May 15 2008;122(10):2315-21. doi:10.1002/ijc.23301

23. Rios P, Bailey HD, Poulalhon C, et al. Parental smoking, maternal alcohol consumption during pregnancy and the risk of neuroblastoma in children. A pooled analysis of the ESCALE and ESTELLE French studies. *International Journal of Cancer*. Dec 2019;145(11):2907-2916. doi:10.1002/ijc.32161

24. Rios P, Bailey HD, Lacour B, et al. Maternal use of household pesticides during pregnancy and risk of neuroblastoma in offspring. A pooled analysis of the ESTELLE and ESCALE French studies (SFCE). Article. *Cancer Causes and Control*. 2017;28(10):1125-1132. doi:10.1007/s10552-017-0944-5

25. Hug K, Grize L, Seidler A, Kaatsch P, Schüz J. Parental occupational exposure to extremely low frequency magnetic fields and childhood cancer: a German case-control study. *Am J Epidemiol*. Jan 1 2010;171(1):27-35. doi:10.1093/aje/kwp339

26. Schuz J, Forman MR. Birthweight by gestational age and childhood cancer. *Cancer Causes & Control*. Aug 2007;18(6):655-663. doi:10.1007/s10552-007-9011-y

27. Schüz J, Weihkopf T, Kaatsch P. Medication use during pregnancy and the risk of childhood cancer in the offspring. *Eur J Pediatr*. May 2007;166(5):433-41. doi:10.1007/s00431-006-0401-z

28. Schuz J, Kaatsch P, Kaletsch U, Meinert R, Michaelis J. Association of childhood cancer with factors related to pregnancy and birth. *International Journal of Epidemiology*. Aug 1999;28(4):631-639. doi:10.1093/ije/28.4.631

29. Parodi S, Merlo DF, Ranucci A, et al. Risk of neuroblastoma, maternal characteristics and perinatal exposures: The SETIL study. *Cancer Epidemiology*. 2014/12/01/ 2014;38(6):686-694. doi:<https://doi.org/10.1016/j.canep.2014.09.007>

30. Hardell L, Dreifaldt AC. Breast-feeding duration and the risk of malignant diseases in childhood in Sweden. *Eur J Clin Nutr*. Mar 2001;55(3):179-85. doi:10.1038/sj.ejcn.1601142

31. Rajaraman P, Simpson J, Neta G, et al. Early life exposure to diagnostic radiation and ultrasound scans and risk of childhood cancer: case-control study. *Bmj*. Feb 10 2011;342:d472. doi:10.1136/bmj.d472

32. Pang D, McNally R, Birch JM, Investig UKCCS. Parental smoking and childhood cancer: results from the United Kingdom Childhood Cancer Study. *British Journal of Cancer*. Feb 2003;88(3):373-381. doi:10.1038/sj.bjc.6600774

33. Buck GM, Michalek AM, Chen CJ, Nasca PC, Baptiste MS. Perinatal factors and risk of neuroblastoma. *Paediatr Perinat Epidemiol*. Jan 2001;15(1):47-53. doi:10.1046/j.1365-3016.2001.00307.x

34. Daniels JL, Olshan AF, Pollock BH, Shah NR, Stram DO. Breast-feeding and neuroblastoma, USA and Canada. *CANCER CAUSES & CONTROL*. JUN 2002;13(5):401-405. doi:10.1023/A:1015746701922

35. Patton T, Olshan AF, Neglia JP, Castleberry RP, Smith J. Parental exposure to medical radiation and neuroblastoma in offspring. *Paediatric and Perinatal Epidemiology*. May 2004;18(3):178-185. doi:10.1111/j.1365-3016.2004.00554.x

36. Daniels JL, Olshan AF, Teschke K, et al. Residential pesticide exposure and neuroblastoma. *Epidemiology*. Jan 2001;12(1):20-7. doi:10.1097/00001648-200101000-00005

37. Olshan AF, De Roos AJ, Teschke K, et al. Neuroblastoma and parental occupation. *Cancer Causes & Control*. 1999;10(6):539-549. doi:10.1023/a:1008998925889

38. Olshan AF, Smith JC, Bondy ML, Neglia JP, Pollock BH. Maternal vitamin use and reduced risk of neuroblastoma. *Epidemiology*. Sep 2002;13(5):575-80. doi:10.1097/00001648-200209000-00014

39. De Roos AJ, Teschke K, Savitz DA, et al. Parental occupational exposures to electromagnetic fields and radiation and the incidence of neuroblastoma in offspring. *Epidemiology*. Sep 2001;12(5):508-517. doi:10.1097/00001648-200109000-00008

40. Kerr MA, Nasca PC, Mundt KA, Michalek AM, Baptiste MS, Mahoney MC. Parental occupational exposures and risk of neuroblastoma: a case-control study (United States). *Cancer Causes Control*. Aug 2000;11(7):635-43. doi:10.1023/a:1008951632482

41. Schwartzbaum JA. INFLUENCE OF THE MOTHERS PRENATAL DRUG CONSUMPTION ON RISK OF NEUROBLASTOMA IN THE CHILD. *American Journal of Epidemiology*. Jun 1992;135(12):1358-1367. doi:10.1093/oxfordjournals.aje.a116247

42. BUNIN GR, WARD E, KRAMER S, RHEE CA, MEADOWS AT. NEUROBLASTOMA AND PARENTAL OCCUPATION. *American Journal of Epidemiology*. 1990;131(5):776-780. doi:10.1093/oxfordjournals.aje.a115568

43. Stavrou EP, Baker DF, Bishop JF. Maternal smoking during pregnancy and childhood cancer in New South Wales: a record linkage investigation. *Cancer Causes Control*. Nov 2009;20(9):1551-8. doi:10.1007/s10552-009-9400-5

44. Heck JE, Lee PC, Wu CK, et al. Gestational risk factors and childhood cancers: A cohort study in Taiwan. *International Journal of Cancer*. Sep 2020;147(5):1343-1353. doi:10.1002/ijc.32905

45. Huang X, Hansen J, Lee P-C, et al. Maternal diabetes and childhood cancer risks in offspring: two population-based studies. *British Journal of Cancer*. 2022/09/10 2022;doi:10.1038/s41416-022-01961-w

46. Volk J, Heck JE, Schmiegelow K, Hansen J. Parental occupational organic dust exposure and selected childhood cancers in Denmark 1968-2016. *Cancer Epidemiol*. Apr 2020;65:101667. doi:10.1016/j.canep.2020.101667

47. Schuz J, Luta G, Erdmann F, et al. Birth order and risk of childhood cancer in the Danish birth cohort of 1973-2010. *Cancer Causes & Control*. Nov 2015;26(11):1575-1582. doi:10.1007/s10552-015-0651-z

48. Contreras ZA, Hansen J, Ritz B, Olsen J, Yu F, Heck JE. Parental age and childhood cancer risk: A Danish population-based registry study. *CANCER EPIDEMIOLOGY*. AUG 2017;49:202-215. doi:10.1016/j.canep.2017.06.010

49. Seppälä LK, Vettenranta K, Leinonen MK, Tommiska V, Madanat-Harjuoja LM. Preterm birth, neonatal therapies and the risk of childhood cancer. *Int J Cancer*. May 1 2021;148(9):2139-2147. doi:10.1002/ijc.33376

50. Mortensen JHS, Øyen N, Fomina T, et al. Supplemental folic acid in pregnancy and childhood cancer risk. *British Journal of Cancer*. 2016/01/01 2016;114(1):71-75. doi:10.1038/bjc.2015.446

51. Feychting M, Floderus B, Ahlbom A. Parental occupational exposure to magnetic fields and childhood cancer (Sweden). *Cancer Causes Control*. Feb 2000;11(2):151-6. doi:10.1023/a:1008922016813

52. Bluhm E, McNeil DE, Cnattingius S, Gridley G, El Ghormli L, Fraumeni JF, Jr. Prenatal and perinatal risk factors for neuroblastoma. *Int J Cancer*. Dec 15 2008;123(12):2885-90. doi:10.1002/ijc.23847

53. Sundh KJ, Henningsen AK, Källen K, et al. Cancer in children and young adults born after assisted reproductive technology: a Nordic cohort study from the Committee of Nordic ART and Safety (CoNARTaS). *Hum Reprod*. Sep 2014;29(9):2050-7. doi:10.1093/humrep/deu143

54. Spector LG, Brown MB, Wantman E, et al. Association of In Vitro Fertilization With Childhood Cancer in the United States. *JAMA Pediatrics*. 2019;173(6):e190392-e190392. doi:10.1001/jamapediatrics.2019.0392

55. McLaughlin CC, Baptiste MS, Schymura MJ, Zdeb MS, Nasca PC. Perinatal risk factors for neuroblastoma. *Cancer Causes & Control*. Apr 2009;20(3):289-301. doi:10.1007/s10552-008-9243-5

56. Johnson KJ, Puumala SE, Soler JT, Spector LG. Perinatal characteristics and risk of neuroblastoma. *Int J Cancer*. Sep 1 2008;123(5):1166-72. doi:10.1002/ijc.23645

57. Williams LA, Sample J, McLaughlin CC, et al. Sex differences in associations between birth characteristics and childhood cancers: a five-state registry-linkage study. *Cancer Causes Control*. Nov 2021;32(11):1289-1298. doi:10.1007/s10552-021-01479-1

58. Contreras ZA, Ritz B, Virk J, Cockburn M, Heck JE. Maternal pre-pregnancy and gestational diabetes, obesity, gestational weight gain, and risk of cancer in young children: a population-based study in California. *Cancer Causes Control*. Oct 2016;27(10):1273-85. doi:10.1007/s10552-016-0807-5

59. Heck JE, Contreras ZA, Park AS, Davidson TB, Cockburn M, Ritz B. Smoking in pregnancy and risk of cancer among young children: A population-based study. *Int J Cancer*. Aug 1 2016;139(3):613-6. doi:10.1002/ijc.30111

60. Johnson CC, Spitz MR. Neuroblastoma: case-control analysis of birth characteristics. *J Natl Cancer Inst*. Apr 1985;74(4):789-92.

61. Spitz MR, Johnson CC. Neuroblastoma and paternal occupation. A case-control analysis. *Am J Epidemiol*. Jun 1985;121(6):924-9. doi:10.1093/oxfordjournals.aje.a114062

62. Schraw JM, Rodriguez KB, Scheurer ME, Foster JH, Lupo PJ. Associations of demographic and perinatal factors with childhood neuroblastoma in Texas, 1995–2011. *Cancer Epidemiology*. 2022/06/01/ 2022;78:102165. doi:<https://doi.org/10.1016/j.canep.2022.102165>

63. Kumar SV, Lupo PJ, Pompeii LA, Danysh HE. Maternal residential proximity to major roadways and pediatric embryonal tumors in offspring. Article. *International Journal of Environmental Research and Public Health*. 2018;15(3)doi:10.3390/ijerph15030505

64. Neglia JP, Smithson WA, Gunderson P, King FL, Singher LJ, Robison LL. Prenatal and perinatal risk factors for neuroblastoma. A case-control study. *Cancer*. Jun 1 1988;61(11):2202-6. doi:10.1002/1097-0142(19880601)61:11<2202::aid-cncr2820611113>3.0.co;2-7

65. Su Q, Sun X, Zhu L, et al. Breastfeeding and the risk of childhood cancer: a systematic review and dose-response meta-analysis. *BMC Med*. Apr 13 2021;19(1):90. doi:10.1186/s12916-021-01950-5

66. Martin RM, Gunnell D, Owen CG, Smith GD. Breast-feeding and childhood cancer: a systematic review with metaanalysis. *INTERNATIONAL JOURNAL OF CANCER*. DEC 20 2005;117(6):1020-1031. doi:10.1002/ijc.21274

67. Onyije FM, Olsson A, Baaken D, et al. Environmental Risk Factors for Childhood Acute Lymphoblastic Leukemia: An Umbrella Review. *Cancers*. Jan 2022;14(2)382. doi:10.3390/cancers14020382

68. Betrán AP, Ye J, Moller AB, Zhang J, Gülmezoglu AM, Torloni MR. The Increasing Trend in Caesarean Section Rates: Global, Regional and National Estimates: 1990-2014. *PLoS One*. 2016;11(2):e0148343. doi:10.1371/journal.pone.0148343

69. Jiang L-L, Gao Y-Y, He W-B, Gan T, Shan H-Q, Han X-M. Cesarean section and risk of childhood leukemia: a systematic review and meta-analysis. *World Journal of Pediatrics*. 2020/10/01 2020;16(5):471-479. doi:10.1007/s12519-020-00338-4

70. Han MA, Storman D, Al-Rammahy H, et al. Impact of maternal reproductive factors on cancer risks of offspring: A systematic review and meta-analysis of cohort studies. *PLoS One*. 2020;15(3):e0230721. doi:10.1371/journal.pone.0230721

71. Williams LA, Richardson M, Spector LG, Marcotte EL. Cesarean Section Is Associated with an Increased Risk of Acute Lymphoblastic Leukemia and Hepatoblastoma in Children from Minnesota. *Cancer Epidemiol Biomarkers Prev*. Apr 2021;30(4):736-742. doi:10.1158/1055-9965.Epi-20-1406

72. Cho CE, Norman M. Cesarean section and development of the immune system in the offspring. *American Journal of Obstetrics and Gynecology*. 2013/04/01/ 2013;208(4):249-254. doi:<https://doi.org/10.1016/j.ajog.2012.08.009>

73. Harder T, Plagemann A, Harder A. Birth weight and risk of neuroblastoma: a meta-analysis. *International Journal of Epidemiology*. Jun 2010;39(3):746-756. doi:10.1093/ije/dyq040

74. Poma PA. Correlation of birth weights with cesarean rates. *Int J Gynaecol Obstet*. May 1999;65(2):117-23. doi:10.1016/s0020-7292(98)00261-6

75. Khan A, Feulefack J, Sergi CM. Pre-conceptional and prenatal exposure to pesticides and pediatric neuroblastoma. A meta-analysis of nine studies. *Environmental Toxicology and Pharmacology*. 2022/02/01/ 2022;90:103790. doi:<https://doi.org/10.1016/j.etap.2021.103790>
